# CapR: revealing structural specificities of RNA-binding protein target recognition using CLIP-seq data

**DOI:** 10.1186/gb-2014-15-1-r16

**Published:** 2014-01-21

**Authors:** Tsukasa Fukunaga, Haruka Ozaki, Goro Terai, Kiyoshi Asai, Wataru Iwasaki, Hisanori Kiryu

**Affiliations:** 1 Department of Computational Biology, Graduate School of Frontier Sciences, the University of Tokyo, Chiba 277-8568, Japan; 2 INTEC Inc, 1-3-3 Shinsuna Koto-ku, Tokyo 136-8637, Japan; 3 Computational Biology Research Center, National Institute of Advanced Industrial Science and Technology, Tokyo 135-0064, Japan; 4 Atmosphere and Ocean Research Institute, University of Tokyo, Chiba 277-8564, Japan

## Abstract

RNA-binding proteins (RBPs) bind to their target RNA molecules by recognizing specific RNA sequences and structural contexts. The development of CLIP-seq and related protocols has made it possible to exhaustively identify RNA fragments that bind to RBPs. However, no efficient bioinformatics method exists to reveal the structural specificities of RBP–RNA interactions using these data. We present CapR, an efficient algorithm that calculates the probability that each RNA base position is located within each secondary structural context. Using CapR, we demonstrate that several RBPs bind to their target RNA molecules under specific structural contexts. CapR is available at https://sites.google.com/site/fukunagatsu/software/capr.

## Background

RNA-binding proteins (RBPs) play integral roles in various post-transcriptional regulatory processes, including the splicing, processing, localization, degradation and translation of RNA molecules [1]. RBPs typically contain a limited set of RNA-binding domains, such as the RNA recognition motif and K homology domain, and they must bind to specific RNA molecules to function. The human genome contains more than 400 annotated RBPs [2]. Although most of these RBPs are still poorly characterized, it is known that the dysfunction of certain RBPs causes severe diseases, such as neurodegenerative disorders, heart failure and cancers [3,4]. RBP–RNA interactions and their specificities are important for understanding the complex gene regulatory networks and the mechanisms of human diseases.

Recent advances in ‘ribonomic’ technologies, such as cross-linking immunoprecipitation high-throughput sequencing (CLIP-seq, also referred to as HITS-CLIP) [5], individual-nucleotide resolution CLIP (iCLIP) [6], and photoactivatable-ribonucleoside-enhanced CLIP (PAR-CLIP) [7], have enabled the study of RBP–RNA interactions, both on a genomic scale and at high resolution. The use of microarrays in the classical RNA-binding protein immunoprecipitation microarray (RIP-Chip) method [8] prevented the precise identification of binding sites. In contrast, CLIP-seq methods bond an RBP and RNAs covalently by ultraviolet cross-linking, collect them by immunoprecipitation and directly sequence the RBP-bound sites of the RNAs. Using these technologies, researchers can identify sequential RNA motifs that are over-represented around the binding sites of each RBP using bioinformatics methods similar to those used for analyzing transcription-factor binding DNA motifs [9]. Such sequential motifs are often very short (up to ten bases), and there are many unbound sites that have the same motif. Thus, sequential motifs alone cannot explain the specificity of RBP–RNA interactions.

RBPs bind to their target RNA molecules by recognizing specific RNA sequences and their structures. Several studies have addressed this issue by calculating the accessibility of RNA regions around the RBP-binding sites [10]. Here, the accessibility of an RNA region is defined by the probability that the region exhibits a single-stranded conformation. Theoretically, the accessibility can be efficiently and exactly calculated using an energy model of RNA secondary structures [11,12]. Double-helical RNAs usually form the A-form helical structure, whose major grooves are too narrow to be accessed by RBPs [13], and Li *et al.* showed that the accessibilities tend to be high around the RBP-bound motif sites by analyzing RIP-Chip data [10]. However, it is not sufficient to consider accessibility alone in analyzing the structure-specific target recognition by RBPs. For example, Vts1p, which is a yeast RBP regulating mRNA stability, binds to its target CNGG sequential motif when it is located within hairpin loops but not when it is located in single-stranded regions or other structures [14,15]. The human FET family of proteins, whose mutations are associated with amyotrophic lateral sclerosis, bind to its target sequential UAN _*n*_Y motif within hairpin loops [16]. Computational methods for calculating the secondary structural contexts of RNA molecules, such as bulge loops, hairpin loops and stems, are required to uncover the characteristics of the RNA structures that are recognized by the RBPs *in vivo*.

**Figure 1:**
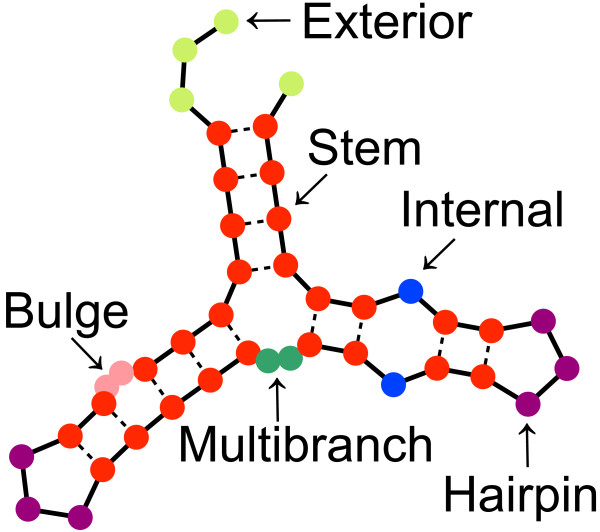
**Visual representation of the six structural contexts.** The six structural contexts are represented by six colors: stems (red), exterior loops (light green), hairpin loops (purple), bulge loops (pink), internal loops (blue) and multibranch loops (green). The unstructured context is the union of the exterior and multibranch loops. These colors are used throughout the paper.

In the present study, we developed an efficient algorithm that calculates the probabilities that each RNA base position is located within each secondary structural context. Six contexts of RNA secondary structures were taken into account, according to the well-established Turner energy model of RNAs [17]. These structures included stems (S), hairpin loops (H), bulge loops (B), internal loops (I), multibranch loops (M) and exterior loops (E) (see Figure 1). We defined a *structural profile* of an RNA base as a set of six probabilities that the base belongs to each context. At present, Sfold [18] is the only software that can calculate a structural profile. Sfold cannot be readily applied to tens of thousands RNA fragments because it uses a statistical sampling method that requires huge sample sizes and computational costs, particularly when analyzing long RNAs or mRNAs. We implemented our efficient algorithm as software named ‘CapR’, which can compute the structural profiles for tens of thousands of long RNAs within a reasonable time by enumerating all the possible secondary structures of the RNAs.

## Results

### Methods overview

We have developed a new algorithm that calculates the structural profiles of any RNA sequence based on the Turner energy model with time complexity *O*(*NW*^2^) [17]. Here, *N* is the input sequence length and *W* is the *maximal span*, which is a given parameter of the maximal length between the bases that form base pairs. The parameter *W* was introduced because considering very long interactions does not improve the accuracy of the secondary structure predictions but does increase the computational costs [19].

Let *x* be an RNA sequence of length *N* and *σ* be a possible secondary structure on *x* without pseudoknots. We refer to a base in *x* as *stem* if it forms a base pair with another base, and represent it using the character S. Single-stranded bases are categorized into five structural contexts, namely, *bulge loop* (represented by B), *exterior loop* (E), *hairpin loop* (H), *internal loop* (I) and *multibranch loop* (M), which are defined as follows. In a secondary structure representation, RNA bases are vertices of polygons whose edges are the RNA backbone or hydrogen bonds, which are shown as solid or dotted lines, respectively, in Figure 1. The exterior loop context is given to single-stranded bases if they do not form polygons. The hairpin loop context is given to single-stranded bases if they form a polygon that has a single hydrogen bond. The bulge and internal loop contexts are given to single-stranded bases if they form a polygon that has two hydrogen bonds, which are connected by a single backbone edge for bulge loops and which are not connected by a single backbone edge for internal loops. Finally, the multibranch loop context is given to single-stranded bases if they form a polygon that has more than two hydrogen bonds. Note that for a given secondary structure *σ*, any base of *x* is unambiguously classified as one of the six structural contexts. Additionally, we define *unstructured* (U) to represent collectively the exterior and multibranch loop contexts.

We assume that the probability distribution of the secondary structures follows the Boltzmann distribution with respect to the Turner energy model [17]. The probability *p*(*i*,*δ*) that a base at position *i* has the structural context *δ*∈{*B*,*E*,*H*,*I*,*M*,*S*} is given by

$$\begin{array}{lll} p(i,\delta ) & =\frac{1}{Z(x)}\underset{\sigma \in \Omega (i,\delta )}{\sum }\exp\left(-\mathrm{\Delta G}(\sigma ,x)/\text{RT}\right)\; & \;\\ Z(x) & =\underset{\sigma \in \Omega_{0}}{\sum }\exp\left(-\mathrm{\Delta G}(\sigma ,x)/\text{RT}\right)\; & \; \end{array}$$

where *ΔG*(*σ*,*x*) is the difference of the Gibbs energies of the given structure *σ* and the structure *σ*_0_ that contains no base pairs, *R* is the gas constant and *T* is the temperature (we used *T*=310.15 K in this study). *Ω*_0_ is the set of all the possible secondary structures of *x*, and *Ω*(*i*,*δ*) is the set of all the possible secondary structures in which the base at position *i* is in the structural context *δ*. Then, the structural profile of *i* is defined as the probabilities of the structural contexts {*p*(*i*,*δ*)|*δ*∈{*B*,*E*,*H*,*I*,*M*,*S*}}. Note that the structural profile satisfies the probability condition $\underset{\delta }{\sum }p(i,\delta )=1$.

Our algorithm efficiently calculates structural profiles by referring to the Rfold model, which is a variant of the stochastic context-free grammar (SCFG) that calculates all the RNA secondary structures without redundancy [20]. In formal language theory, the RNA secondary structures without pseudoknots are modeled by SCFG [21]. While the state transition rules of the Rfold model contain seven non-terminal symbols, our algorithm associated them with the six structural contexts. The details of the algorithm, which is a variant of the inside-outside algorithm of SCFG, are given in the Materials and methods section.

### Influence of the maximal span and the GC content on the structural profile calculations

Before we investigated the structure-specific target recognition by RBPs, we evaluated the performance of CapR. Because we introduced the maximal span *W*, we needed to investigate an appropriate range for this parameter. Because GC content is known to affect the RNA secondary structures, its effect was also analyzed.

**Figure 2:**
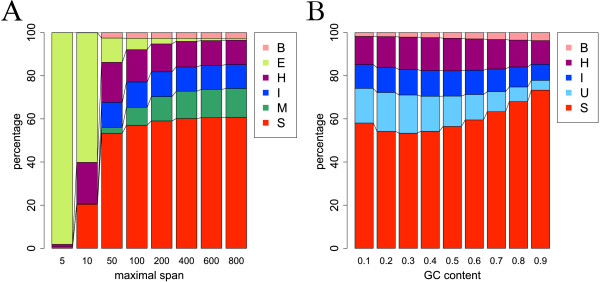
**Dependence of the structural profiles on the maximal span*W* and GC content.(A)** Dependence of the structural profiles on the maximal span *W*. The *x*-axis represents the maximal span *W*. The *y*-axis represents the averaged *p*(*i*,*δ*) over all the nucleotides. **(B)** Dependence of the structural profiles on the GC content. The *x*-axis represents the GC content. The *y*-axis represents the averaged *p*_*δ*_(*i*) over all the nucleotides. The unstructured context is represented by light blue. B, bulge loop; E, exterior loop; H, hairpin loop; I, internal loop; M, multibranch loop; S, stem; U, unstructured.

To investigate the dependence on the maximal span *W*, we applied CapR to 1,000 random RNA sequences of 2,000 nucleotides with a fixed GC content (GC = 0.5). Figure 2A shows how the proportions of the calculated structural profiles depend on *W*. As expected, if *W* is small, the predictions are dominated by exterior loops because few bases form base pairs under this condition. Whereas the probabilities for bulge loops, hairpin loops, internal loops and stems are relatively stable for *W*≥100, the exterior loop probabilities monotonically decrease and the multibranch loop probabilities monotonically increase with increasing *W*. This is because at large *W* new base pairs form in exterior loops and exterior loops turn into multibranch loops. On the other hand, the probabilities of the unstructured context, which collectively represents the exterior and multibranch loop contexts, are insensitive to *W* (Additional file 1: Figure S1). Therefore, the unstructured context can be adopted instead of the exterior and multibranch loop contexts to avoid the influence of the parameter *W*, if a discrimination of the two contexts is not critical.

Although Kiryu *et al.* revealed the dependence of the accessibilities on the GC content [12], the dependence of structural profiles on the GC content has not been investigated. We investigated the dependence on the GC content by applying CapR to 1,000 random RNA sequences of 2,000 nucleotides with a fixed maximal span (*W* = 100). Figure 2B shows how the proportions of the computed structural profiles depend on the GC content. The stem probability is high and the unstructured probability is low with a high GC content, probably because the energy of the G-C pairs is larger than that of the A-U pairs and palindromic sequences are more likely to occur in the high-GC background. This result suggests that users should carefully interpret the results when analyzing RNAs with biased GC content.

### Performance of CapR

**Figure 3:**
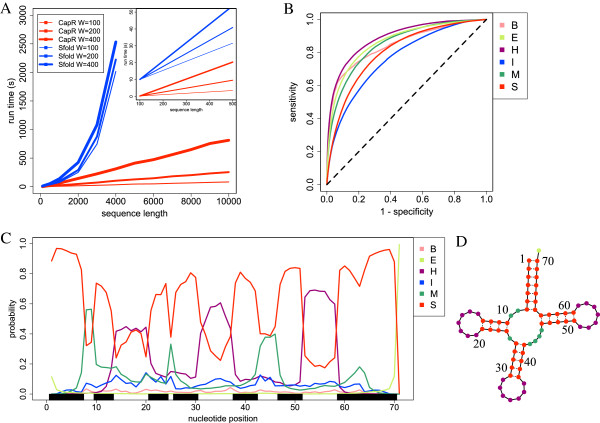
**Performance of CapR.(A)** Computational run-times for different values of maximal span *W* and sequence length *N*. The *x*-axis represents the sequence length *N*. The *y*-axis represents the computational run-time. **(B)** The receiver operating characteristic curve for each loop context. The *x*-axis represents 1-specificity and the *y*-axis represents the sensitivity. The specificity and sensitivity are defined as true positive/(true positive + false negative) and true negative/(true negative + false positive), respectively. **(C)** The structural profiles of tRNAs. The *x*-axis represents the nucleotide positions from 5^′^ to 3^′^. The *y*-axis represents averaged probabilities that each base belongs to each structural context across all tRNA genes in the Rfam dataset [22]. The black boxes represent the nucleotides annotated as stem in Rfam. **(D)** tRNA cloverleaf structure annotated in Rfam. B, bulge loop; E, exterior loop; H, hairpin loop; I, internal loop; M, multibranch loop; S, stem.

We evaluated the speed of CapR by comparing its computational run-time with that of Sfold. The input sequences were generated randomly with equal probabilities of A, C, G and U. For Sfold, the number of sampled structures was set to its default value (1,000). The computation was performed on an AMD Opteron 6276 2.3 GHz with 1 GB memory. Figure 3A shows the computational run-times, which depended on the maximal span *W* and sequence lengths. In all cases, CapR was much faster than Sfold. Sfold could not run for *N*≥4,000 while CapR did for *N*=10,000. These results show that CapR can compute structural profiles for long RNAs within a reasonable time.

Next, we evaluated the accuracy of the structural profiles computed by CapR using 8,775 RNA genes that have experimentally validated secondary structure annotations in the Rfam database [22]. We set *W*=800 to allow for stem-forming of the base pairs with the longest distance observed in the Rfam dataset. To estimate the accuracy of the structural profiles, we calculated the area under the receiver operating characteristic curve (AUROC) for each structural context. Briefly, the AUROC is high if the probability *p*(*i*,*δ*) for the structural context *δ* annotated in Rfam is high.

Table 1 and Figure 3B show the AUROC values and the receiver operating characteristic curves, respectively. The AUROC value for each structural context was larger than 0.75, indicating that the computed structural profiles were very consistent with the Rfam annotation. For example, the structural profile of transfer RNAs (tRNAs), whose secondary structures are well characterized, is shown in Figure 3C. Each line represents averaged probabilities that each base belongs to each structural context across all tRNA genes in the Rfam dataset. Probabilities of the stem, hairpin loop, multibranch loop and exterior loop contexts were high at the corresponding parts of the tRNA cloverleaf structure (Figure 3D). Calculated structural profiles are interpreted by considering that stem probabilities tend to be overestimated by the Turner energy model. In the tRNA example, the calculated stem probabilities were slightly higher than the multibranch loop probabilities at positions 25, 43 and 44, which are annotated as multibranch loops in Rfam.

**Table 1:** AUC score of each structural context

**Software**	**Bulge**	**Exterior**	**Hairpin**	**Internal**	**Multibranch**	**Stem**
CapR	0.847	0.866	0.890	0.765	0.852	0.805
Sfold	0.842	0.817	0.890	0.769	0.853	0.804

Finally, the same analysis was conducted using Sfold, and the accuracies of the structural profiles predicted by CapR and Sfold were compared. The accuracies of CapR were comparable to those of Sfold (Table 1).

### Datasets and methods used in the CLIP-seq data analysis

Because it was shown that CapR is accurate in calculating structural profiles of RNA molecules, we applied it to several CLIP-seq datasets to reveal the structural specificities of RBP–RNA interactions. For the subsequent analyses, we downloaded CLIP-seq data of RBP-bound RNAs from the doRina database [23], and selected ten RBPs: GLD-1 (nematode), QKI (human), Pum2 (human), SRSF1 (human), Nova (mouse), Lin28A (mouse), FXR1 (human), FXR2 (human), FMR1_7 (human) and FMR1_1 (human) [7,24-28] (refer to Materials and methods for the criteria for the data selection). FMR1_7 and FMR1_1 are two splicing isoforms of FMR1. RBPs with two known sequential motifs (FXR1, FXR2, FMR1_7 and FMR1_1) were analyzed separately for each of the motifs. Hereafter, these cases are represented by the protein names with their sequential motifs: FXR1(ACUK), FXR1(WGGA), FXR2(ACUK), FXR2(WGGA), FMR1_7(ACUK), FMR1_7(WGGA), FMR1_1(ACUK) and FMR1_1(WGGA).

We created one positive dataset and two negative datasets for each of these 14 cases. The positive dataset was a collection of transcribed sequences of ±2,000 nucleotides around each RBP-bound site. The RBP-bound sites were defined as sites of sequential motifs within the CLIP-seq peak regions. The two negative datasets are referred to as the unbound and shuffled datasets. The unbound dataset was a collection of transcribed sequences of ±2,000 nucleotides around a sequential motif site that was in the same transcriptional unit and within ±1,000 nucleotides of any RBP-bound site, but was not an RBP-bound site. In short, this dataset represents the sequential motif sites that are transcribed but unbound by the RBP. The shuffled dataset was generated by randomly shuffling each of the upstream and downstream sequences of each RBP-bound site by preserving nucleotide di-nucleotide frequencies for every sequence in the positive dataset. Thus it represents the sequential motif sites flanked by sequences with preserved sequence compositions. The details of the datasets are described in the Materials and methods section.

We calculated the structural profiles of the positive, unbound and shuffled datasets for each of the RBPs (*W*=200). Then, to evaluate the structural contexts that are significant in the positive dataset statistically, we defined a *P* score as follows. First, we calculated a *P* value using the one-sided Wilcoxon–Mann–Whitney test for each side for each position. Second, we selected the smaller *P* value of the two hypotheses and transformed it into − log10*P*, which we designated the *P* score. Third, if a *P* score was calculated under the hypothesis that each context probability of the positive dataset was smaller than that of the negative dataset, we changed the sign of the *P* score. For example, a large positive *P* score indicates that the probability of that structural context is significantly larger in the positive dataset. Finally, the two *P* scores calculated for the two negative datasets were compared for each position, and the smaller *P* score was taken (if one *P* score was positive and the other was negative, we used 0 instead of the two *P* scores). Note that the Bonferroni correction was used for multiple testing. To avoid the effects of the artificial value selection for the parameter *W*, we used the unstructured context instead of the exterior and multibranch loop contexts in the following analysis. We confirmed that the choice of *W* actually did not affect the results (Additional file 1: Figure S2).

### Specific RNA structural contexts recognized by RNA-binding proteins

**Figure 4:**
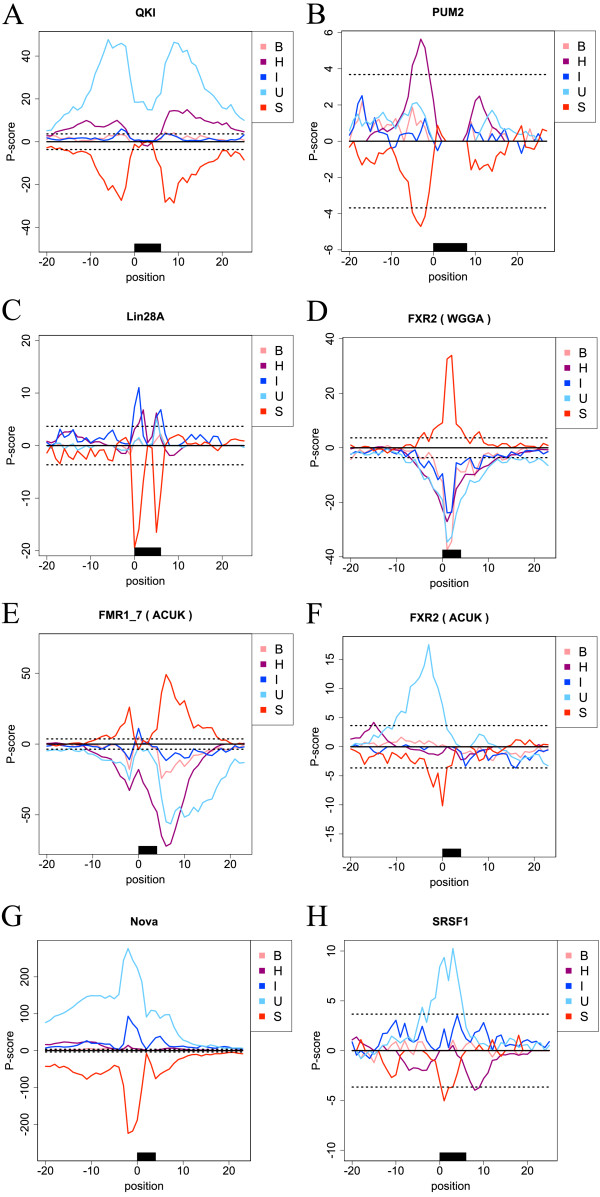
**The distribution of the*P* scores for each RNA-binding protein.** The *x*-axis represents the nucleotide positions and the *y*-axis represents the *P* score of ±20 bases around the sequential motif site. The position 0 denotes the start position of the sequential motif. Positive *P* scores for each structural context indicate that the positions tend to prefer the structural context. The black box represents the sequential motif site. The dotted lines show the corrected significance levels of the Bonferroni correction (*α*=0.05). The panels represent the distribution of *P* scores for **(A)** QKI, **(B)** Pum2, **(C)** Lin28A, **(D)** FXR2(WGGA), **(E)** FMR1_7(ACUK), **(F)** FXR2(ACUK), **(G)** Nova and **(H)** SRSF1. B, bulge loop; H, hairpin loop; I, internal loop; S, stem; U, unstructured.

We investigated the preferred RNA structural contexts for each RBP and revealed that most RBPs prefer a specific structural context (Figure 4 and Additional file 1: Figure S3). Our method was robust regarding the selection of the negative datasets, because selecting the larger *P* scores did not affect the results overall (Additional file 1: Figures S4 and S5). Among the 14 cases analyzed, six cases showed a preference for the unstructured context (GLD-1, QKI, SRSF1, Nova, FXR1(ACUK) and FXR2(ACUK)). Except for Nova, the RBP-bound sites tended to form the unstructured context, but did not show preferences for the bulge, internal or hairpin loop contexts (Figure 4A and Additional file 1: Figure S3). It should be noted that these results could not be obtained by analyzing the accessibility alone, which does not discriminate between these non-stem contexts.

Pum2 showed a preference for the hairpin loop context (Figure 4B). To our knowledge, this is the first report of the structural preference for the hairpin loop context by Pum2, which is known to be involved in germ cell development [29]. Lin28A showed preferences for the hairpin and internal loop contexts (Figure 4C). Lin28A is known to inhibit the maturation of let-7 miRNAs and the translation of mRNAs that are destined for the endoplasmic reticulum [27]. The specificity of Lin28A to the hairpin loop context is consistent with the previous study [27]. In addition, our result is the first to suggest that Lin28A prefers the internal loop context in mRNA binding, and Lin28A has been reported to bind to the internal loop of let-7 miRNAs [27].

FXR1(WGGA), FXR2(WGGA) and FMR1_7(WGGA) showed preferences for the stem context (Figure 4D and Additional file 1: Figure S3), although RBPs were considered to be unlikely to be bound to the stem regions of RNAs as already mentioned. These three RBPs (and FMR1_1) are members of the FMRP family and are known to be responsible for the fragile X syndrome. Darnell *et al.* showed that FMRP-bound WGGA sites tend to form a G-quadruplex, which is composed of guanine-rich sequences forming a four-stranded RNA structure [30]. We suppose that the preference for the stem contexts could reflect the tendency that these family members recognize the G-quadruplex; however, this should be investigated further as currently our energy model and grammar cannot deal with G-quadruplexes.

FMR1_7(ACUK) showed preferences for the internal and bulge loop contexts (Figure 4E). To our knowledge, this is the first report of the structural specificities of FMR1. In contrast, FXR2(ACUK), where FXR2 is a homolog of FMR1, preferred neither the internal nor bulge loop context (Figure 4F). FMR1_7 has an exon insertion in its K homology domain that recognizes the ACUK sequential motifs [28]. This insertion appears to underlie the differences in the structural specificity between FMR1_7(ACUK) and FXR2(ACUK).

### Positional preferences in the RNA structure recognition by RNA-binding proteins

The present understanding of the structural specificities of RBP–RNA interactions overlooks structures of the flanking sequences of RBP-bound sites. Therefore, we investigated the secondary structures not only of the RBP-bound sites but also of their flanking sequences. In fact, the positions with the highest *P* scores were not within the RBP-bound sites in some RBPs. QKI (Figure 4A), Nova (Figure 4G) and SRSF1 (Figure 4H) preferred the unstructured context. High *P* scores were observed within the RBP-bound sites for SF2ASF, whereas they were observed in the flanking and upstream sequences for QKI and Nova, respectively. These results suggest that RBPs also recognize specific structures existing outside of sequential motif sites, and CapR can uncover these positional preferences from ribonomic datasets.

**Figure 5:**
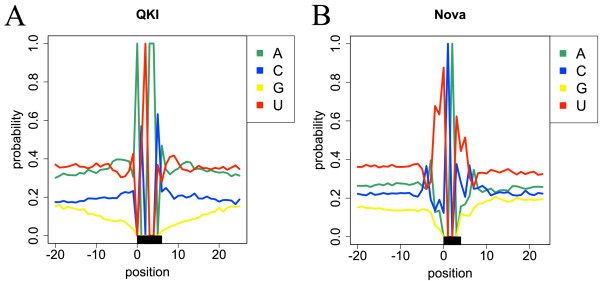
**The nucleotide compositions around the RBP-bound sites.** The nucleotide compositions of ±20 bases around the RBP-bound sites for **(A)** QKI and **(B)** Nova. The *x*-axis represents the nucleotide position and the *y*-axis is the probability of each nucleotide. The black box represents the sequential motif site.

**Figure 6:**
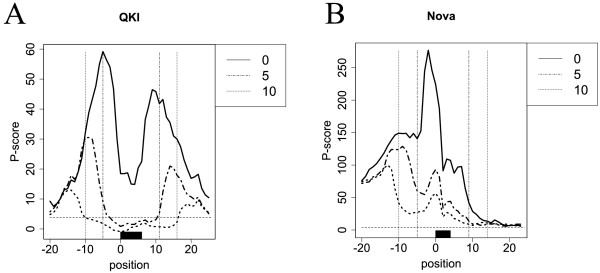
**Comparison of*P* scores of the positive datasets with*P* scores of the shuffled and partially shuffled datasets.** In the legend of this figure, ‘0’, ‘5’ and ‘10’ represents the shuffled, the partially shuffled (±5) and the partially shuffled (±10) datasets, respectively. The *x*-axis represents the nucleotide position and the *y*-axis is the *P* score of **(A)** QKI and **(B)** Nova. The black boxes are the RBP-bound sites, and the horizontal dotted lines the corrected significance levels of the Bonferroni correction. The vertical dotted lines indicate the ±5 or 10 nucleotides of RBP-bound sites. RBP, RNA-binding protein.

Figure 5A,B shows the nucleotide compositions around the RBP-bound sites of QKI and Nova. The flanking sequences of QKI-bound sites were guanine poor, whereas those of Nova-bound sites were uracil rich. Because sequences with low GC content tend to form an unstructured context, the aforementioned positional preferences could be generated by the biased nucleotide compositions. To address this possibility, we investigated the relations between the nucleotide compositions and structural specificities in the flanking sequences. We generated partially shuffled datasets by randomly shuffling sequences outside of the ±5 or 10 nucleotides of the RBP-bound sites with preserving di-nucleotide frequencies, and compared their structural profiles with those of the positive datasets using the Wilcoxon–Mann–Whitney test. Then, the *P* scores for the shuffled and partially shuffled datasets were compared (Figure 6A,B). For QKI, whereas the shuffled dataset had positional preferences in the flanking sequences, the partially shuffled datasets had no significant preferences. This means that the structural specificities of QKI could be generated by the biased nucleotide compositions in the flanking sequences. For Nova, the partially shuffled datasets still had significant *P* scores upstream of the RBP-bound sites. Therefore, the nucleotide compositions in the flanking sequences alone cannot generate the positional specificities of Nova, that is, sequences in distant regions could also contribute to the position-specific RNA binding of Nova. The nucleotide compositions around the RBP-bound sites and the analyses of the partially shuffled datasets of other RBPs are described in Additional file 1: Figures S6 and S7, respectively.

## Discussion

In this study, we developed an efficient algorithm that calculates the structural profiles of RNAs, and implemented it as CapR. It is the fastest software that can be applied to tens of thousands of long RNAs.

Using CapR, we investigated structural specificities of RBP target recognition using several CLIP-seq datasets. Our analysis revealed that most RBPs prefer specific structural contexts and some RBPs show positional preferences in their structural recognition. These findings could provide insights into the mechanisms of diseases involving RBPs. FMR1_7, where FMR1 is a causative gene of the fragile X syndrome, was revealed to bind specifically to internal and bulge loops. The observed structural specificity raises the possibility that disruption of the internal or bulge loop structures within the target sites of FMR1_7 may cause this disease. On the other hand, the structural specificities of Nova were revealed to be affected by the sequences of distant regions. This means that a mutation of a nucleotide distant from the RBP-bound sites can cause changes to the secondary structures around the RBP-bound sites. Because some disease-associated single nucleotide polymorphisms in non-coding regions are reported to affect RNA secondary structures [31,32], CapR could also contribute to exploring disease mechanisms behind such polymorphisms.

**Figure 7:**
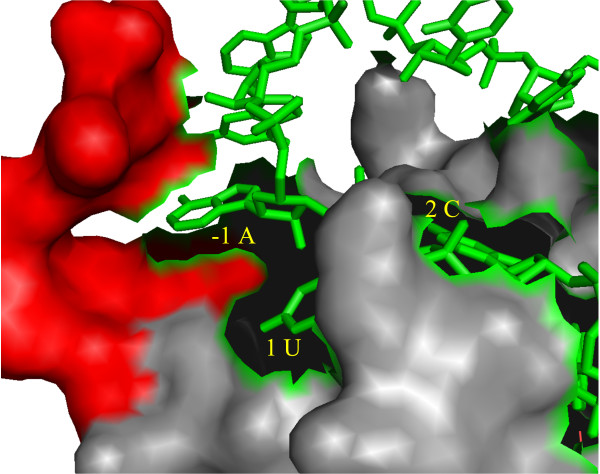
**Co-crystal structure of Nova and the target RNA.** This figure was generated using Pymol. The ten amino acids of the C-terminal tail are shown in red. RNA is represented by green sticks. The positions and the nucleotides are shown in yellow. Position 1 is the start position of the sequential motif.

It has been shown that the secondary structures around the target sites of small interfering RNAs (siRNAs) and miRNAs influence their activities [33,34]. Kiryu *et al.* showed that the activity of an siRNA depends on the accessibility of the 3 ^′^ end of the siRNA target site, and Marin *et al.* showed that the 3 ^′^ end of an miRNA target site is more accessible than the other positions [12,35]. As supported by the X-ray crystal structure of the guide-strand-containing Argonaute [36], these positional tendencies in the accessibility can reflect the kinetic aspects of the siRNA and miRNA binding mechanisms. We hypothesize that the positional preferences of RBPs discovered in this study also reflect the kinetic aspects of the RBP–RNA interactions. For example, Nova had a positional preference for upstream of the sequential motif site in the unstructured context recognition. In fact, the co-crystal structure of human Nova with the target RNA (PDBID: 1EC6) [37] showed that the area upstream of the sequential motif site interacts with the C-terminal amino acids of Nova [38] (see Figure 7; note that the CLIP-seq data were for a highly similar ortholog, mouse Nova). In addition, the deletion of these C-terminal amino acids inhibits the RNA binding function of Nova [39]. Therefore, the positional preference does likely reflect the kinetic aspects of the RNA binding function of Nova. We argue that this example demonstrates the potential power of ribonomic analysis.

Three future perspectives are envisioned based on the present study. The first perspective is to estimate the sequential and structural specificities simultaneously. Throughout this study, we focused on the RBPs with known and well-defined sequential motifs. Nonetheless, for several RBPs, no such sequential motifs have been identified (for example, FET binds to a highly flexible UAN_*n*_Y motif within the hairpin context [16]). To examine the binding specificities of these RBPs, CapR needs to be extended. The second perspective is prediction of RBP-bound sites. Li *et al.* showed that prediction of RBP-bound RNAs *in vivo* was improved by a motif-finding algorithm that considers accessibility [10]. Thus, consideration of structural profiles may also improve the prediction of RBP-bound sites *in vivo*, although we did not directly show this in the present study. Further investigation is necessary for evaluating whether discrimination of RBP-binding sites from a background sequence would be improved using the structural specificities of RBP target recognition. Other factors or subcellular localizations also need to be considered. The third perspective is application of CapR to functional RNAs. For example, the kissing hairpin, which is a hairpin–hairpin interaction that stabilizes RNA structures [40], may be predicted accurately using CapR because CapR enables the calculation of the hairpin loop probabilities. Another target would be small nucleolar RNAs (snoRNAs), where the detection algorithms still have room for improvement [41]. Because snoRNAs are characterized by specific internal loops, they may also be predicted accurately by taking advantage of the accurate calculation of internal loop probabilities by CapR.

## Conclusions

We developed a highly efficient algorithm that calculates the probabilities that each RNA base position is located within each secondary structural context for tens of thousands of RNA fragments. The algorithm was implemented as software named CapR and was applied to the CLIP-seq data of various RBPs. Our algorithm demonstrated that several RBPs bind to their target RNA molecules under specific structural contexts. For example, FMR1, which is an RBP responsible for the fragile X syndrome, was found to bind specifically to the internal and bulge loops of RNA. Another example is Nova, a neuron-specific RBP related to a paraneoplastic neurologic disorder, which showed positional preference in the structural contexts of binding targets.

Secondary structures are known to be essential for the molecular functions of RNA. As large-scale, high-throughput approaches are becoming more popular in studying RNAs and RBPs, our algorithm will contribute to the systematic understanding of RNA functions and structure-specific RBP–RNA interactions.

## Materials and methods

### Rfold model

The state transition rules of the Rfold model are given by

$$\begin{array}{lcrr} \text{Outer} & \to & \varepsilon |\text{Outer}\cdot a|\text{Outer}\cdot \text{Stem} & \\ \text{Stem} & \to & b_{<}\cdot \text{Stem}\cdot b_{>}|b_{<}\cdot \text{StemEnd}\cdot b_{>} & \\ \text{StemEnd} & \to & s_{n}|s_{m}\cdot \text{Stem}\cdot s_{n}(m+n>0)|\text{Multi} & \\ \text{Multi} & \to & a\cdot \text{Multi}|\text{MultiBif} & \\ \text{MultiBif} & \to & \text{Multi1}\cdot \text{Multi2} & \\ \text{Multi1} & \to & \text{MultiBif}|\text{Multi2} & \\ \text{Multi2} & \to & \text{Multi2}\cdot a|\text{Stem} & \end{array}$$

where *ε* represents the null terminal symbol, *a* is an unpaired nucleotide character, *s*_*k*_ is an unpaired base string of length *k* and (*b*_<_, *b*_>_) is a base pair. There are seven non-terminal symbols: Outer, Stem, StemEnd, Multi, MultiBif, Multi1 and Multi2. Outer emits exterior bases. Stem emits all the base pairs. StemEnd represents the end of each stem from which a hairpin loop (StemEnd→*s*_*n*_), and internal and bulge loop (StemEnd→*s*_*m*_·Stem·*s*_*n*_(*m*+*n*>0)), or a multibranch loop (StemEnd→Multi) is emitted. Multi represents a complete multibranch loop. Multi1, Multi2 and MultiBif represent parts of a multibranch loop structure that contains one or more, exactly one, and two or more base pairs in the loop, respectively. Based on this grammar, the structural profiles are calculated by using a variant of the inside-outside algorithm for SCFG. First, we give an illustrative example to show how to calculate the internal loop probabilities from the inside and outside variables *α*_*s*_(*i*,*j*) and *β*_*s*_(*i*,*j*) (*i*,*j*=0,…,*N*, *s*∈{Outer,Stem,StemEnd,Multi,MultiBif,Multi1,Multi2 }). In the subsequent section, we completely describe how to calculate structural profiles.

### Algorithm for calculating internal loop probabilities

**Figure 8:**
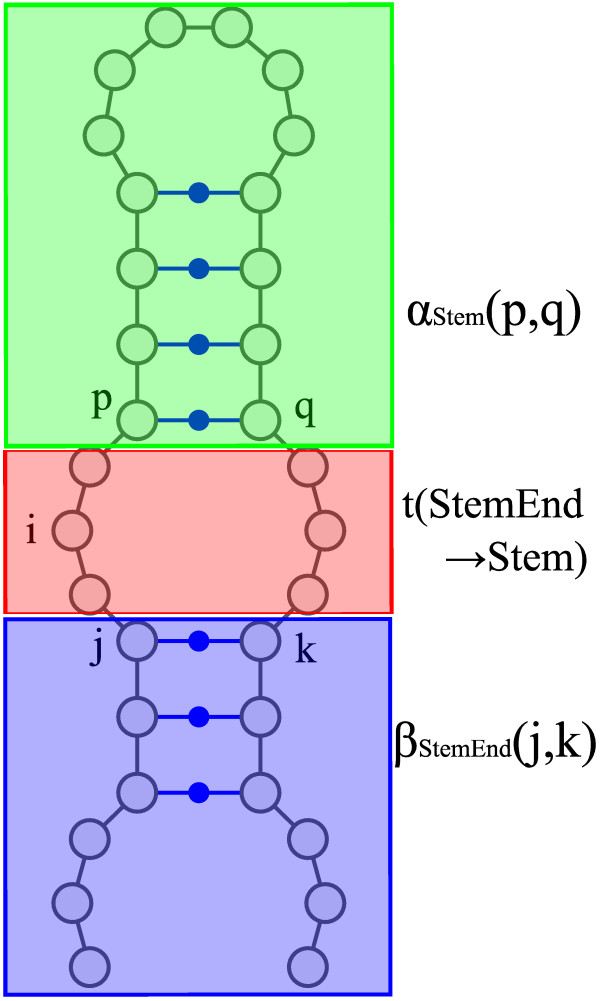
**Schematic illustration of calculation of internal loop probability.** This figure shows the transition patterns that emit an internal loop. This figure was generated by modifying the output of VARNA [42].

When a base at position *i* has an internal loop context, the base *i* is caught in two base pairs, (*j*, *k*) and (*p*, *q*) where *j*≤*p*≤*q*≤*k* (Figure 8). Then, the outside structure of base pair (*j*, *k*) and the inside structure of base pair (*p*, *q*) may take arbitrary structures. The sums of Boltzmann weights of all patterns of the outside structure of base pair (*j*, *k*) and the inside structure of base pair (*p*, *q*) are represented by outside variable *β*_*StemEnd*_(*j*,*k*−1) and inside variable *α*_*Stem*_(*p*−1,*q*), respectively. Therefore, Boltzmann weights that the base *i* is caught in two base pairs (*j*, *k*) and (*p*, *q*) are obtained by the multiplication of *β*_*StemEnd*_(*j*,*k*−1), the score for transition *StemEnd* (*j*,*k*−1)→*Stem*(*p*−1,*q*), and *α*_*S*_*tem*(*p*−1,*q*). Here, we sum these Boltzmann weights for all combinations of base pairs (*j*, *k*) and (*p*, *q*). Finally, we obtain *p*(*i*,*I*) by dividing the sum by the partition function.

The calculation formulas are given by:

$$\begin{array}{lcrr} w(i,I) & = & w_{\text{InternalLeft}}(i,I)+w_{\text{InternalRight}}(i,I) & \\ w_{\text{InternalLeft}}(i,I) & = & \overset{i}{\underset{j=\text{max}(1,i-W)}{\sum }}\overset{\text{min}(n,j+W)}{\underset{k=i+1}{\sum }}\overset{\text{min}(j+C+1,k-1)}{\underset{p=i+1}{\sum }}\overset{k}{\underset{q=\text{max}(p+4,k-C-p+j-1)}{\sum }} & \\ & & \;\beta_{\text{StemEnd}}(j,k-1)\cdot \alpha_{\text{Stem}}(p-1,q)\cdot t(\text{StemEnd}\to (\text{Interior})\to \text{Stem}) & \\ w_{\text{InternalRight}}(i,I) & = & \overset{i}{\underset{j=\text{max}(1,i-W)}{\sum }}\overset{\text{min}(n,j+W)}{\underset{k=i+1}{\sum }}\overset{\text{min}(j+C+1,i-1)}{\underset{p=j+1}{\sum }}\overset{i}{\underset{q=\text{max}(p+4,k-C-p+j-1)}{\sum }} & \\ & & \;\beta_{\text{StemEnd}}(j,k-1)\cdot \alpha_{\text{Stem}}(p-1,q)\cdot t(\text{StemEnd}\to (\text{Interior})\to \text{Stem}) & \\ p(i,I) & = & w(i,I)/Z(x) & \end{array}$$

where *t*(*s*→*s*^′^) is the score for transition *s*→*s*^′^ and *C* is the maximal length of the internal and bulge loops. Many software programs, including RNAfold [43], adopt this parameter. In this study, following the default setting of RNAfold, we set *C*=30.

### Algorithms for calculating the structural profile

#### The inside algorithm and the outside algorithm

To calculate the inside and outside variables, we developed a variant of the inside-outside algorithm corresponding to the Rfold model. The inside algorithm is described as follows:

$$\begin{array}{lcrr} \alpha_{\text{Stem}}(i,j) & = & \sum \left\{\begin{array}{l} \alpha_{\text{Stem}}(i+1,j-1)\cdot t(\text{Stem}\to \text{Stem})\\ \alpha_{\text{Stem}}(i+1,j-1)\cdot t(\text{Stem}\to \text{StemEnd}) \end{array}\right) & \\ \alpha_{\text{Multibif}}(i,j) & = & \sum \left\{\begin{array}{l} \alpha_{\text{Multi1}}(i,k)\cdot \alpha_{\text{Multi2}}(k,j)\cdot t(\text{MultiBif}\to \text{Multi1}\cdot \text{Multi2})\\ \text{for}\;i<k<j \end{array}\right) & \\ \alpha_{\text{Multi2}}(i,j) & = & \sum \left\{\begin{array}{l} \alpha_{\text{Stem}}(i,j)\cdot t(\text{Multi2}\to \text{Stem})\\ \alpha_{\text{Multi2}}(i,j-1)\cdot t(\text{Multi2}\to \text{Multi2}) \end{array}\right) & \\ \alpha_{\text{Multi1}}(i,j) & = & \sum \left\{\begin{array}{l} \alpha_{\text{Multi2}}(i,j)\cdot t(\text{Multi1}\to \text{Multi2})\\ \alpha_{\text{MultiBif}}(i,j)\cdot t(\text{Multi1}\to \text{MultiBif}) \end{array}\right) & \\ \alpha_{\text{Multi}}(i,j) & = & \sum \left\{\begin{array}{l} \alpha_{\text{Multi}}(i+1,j)\cdot t(\text{Multi}\to \text{Multi})\\ \alpha_{\text{MultiBif}}(i,j)\cdot t(\text{Multi}\to \text{MultiBif}) \end{array}\right) & \\ \alpha_{\text{StemEnd}}(i,j) & = & \sum \left\{\begin{array}{l} t(\text{StemEnd}\to (\text{Hairpin}))\\ \alpha_{\text{Stem}}(i^{\prime },j^{\prime })\cdot t(\text{StemEnd}\to (\text{Interior})\to \text{Stem})\\ \text{for}\;i\leq i^{\prime }\leq j^{\prime }\leq j,0<(j-j^{\prime })+(i^{\prime }-i)\leq C\\ \alpha_{\text{Multi}}(i,j)\cdot t(\text{StemEnd}\to \text{Multi}) \end{array}\right) & \\ \alpha_{\text{Outer}}(i) & = & \sum \left\{\begin{array}{l} 1\;\text{if}\;j=0\\ \alpha_{\text{Outer}}(i-1)\cdot t(\text{Outer}\to \text{Outer})\\ \alpha_{\text{Outer}}(k)\cdot \alpha_{\text{Stem}}(k,i)\cdot t(\text{Outer}\to \text{Outer}\cdot \text{Stem})\\ \text{for}\;(i-W)<k<i \end{array}\right) & \end{array}$$

The outside algorithm is described as follows:

$$\begin{array}{lcrr} \beta_{\text{Outer}}(i) & = & \sum \left\{\begin{array}{l} 1\;\text{if}\;i=N\\ \beta_{\text{Outer}}(i+1)\cdot t(\text{Outer}\to \text{Outer})\\ \alpha_{\text{Stem}}(i,k)\cdot \beta_{\text{Outer}}(k)\cdot t(\text{Outer}\to \text{Outer}\cdot \text{Stem})\\ \text{for}\;i<k<i+W \end{array}\right) & \\ \beta_{\text{StemEnd}}(i,j) & = & \beta_{\text{Stem}}(i-1,j+1)\cdot t(\text{Stem}\to \text{StemEnd}) & \\ \beta_{\text{Multi}}(i,j) & = & \sum \left\{\begin{array}{l} \beta_{\text{StemEnd}}(i,j)\cdot t(\text{StemEnd}\to \text{Multi})\\ \beta_{\text{Multi}}(i-1,j)\cdot t(\text{Multi}\to \text{Multi}) \end{array}\right) & \\ \beta_{\text{Multi1}}(i,j) & = & \sum \left\{\begin{array}{l} \beta_{\text{MultiBif}}(i,k)\cdot \alpha_{\text{Multi2}}(j,k)\cdot t(\text{MultiBif}\to \text{Multi1}\cdot \text{Multi2})\\ \text{for}\;j<k<(i+W) \end{array}\right) & \\ \beta_{\text{Multi2}}(i,j) & = & \sum \left\{\begin{array}{l} \beta_{\text{Multi2}}(i,j+1)\cdot t(\text{Multi2}\to \text{Multi2})\\ \beta_{\text{Multi1}}(i,j)\cdot t(\text{Multi1}\to \text{Multi2})\\ \beta_{\text{MultiBif}}(k,j)\cdot \alpha_{\text{Multi1}}(k,i)\cdot t(\text{MultiBif}\to \text{Multi1}\cdot \text{Multi2})\\ \text{for}\;(j-W)<k<i \end{array}\right) & \\ \beta_{\text{MultiBif}}(i,j) & = & \sum \left\{\begin{array}{l} \beta_{\text{Multi1}}(i,j)\cdot t(\text{Multi1}\to \text{MultiBif})\\ \beta_{\text{Multi}}(i,j)\cdot t(\text{Multi}\to \text{MultiBif}) \end{array}\right) & \\ \beta_{\text{Stem}}(i,j) & = & \sum \left\{\begin{array}{l} \alpha_{\text{Outer}}(i)\cdot \beta_{\text{Outer}}(j)\cdot t(\text{Outer}\to \text{Outer}\cdot \text{Stem})\\ \beta_{\text{StemEnd}}(i^{\prime },j^{\prime })\cdot t(\text{StemEnd}\to (\text{Interior})\to \text{Stem})\\ \text{for}\;i^{\prime }\leq i<j\leq j^{\prime },0<(i-i^{\prime })+(j-j^{\prime })\leq C\\ \beta_{\text{Multi2}}(i,j)\cdot t(\text{Multi2}\to \text{Stem})\\ \beta_{\text{Stem}}(i-1,j+1)\cdot t(\text{Stem}\to \text{Stem}) \end{array}\right) & \end{array}$$

The original computational complexity of both algorithms is *O*(*NW*^3^); because we adopted the parameter *C*, it becomes *O*(*NW*^2^) as described below.

#### Calculation of the structural profile

We calculate the structural profiles from the inside and outside variables computed by the inside-outside algorithm. The calculation formula is described as follows:

$$\begin{array}{lcrr} Z & = & \alpha_{O}(N) & \\ p(i,B) & = & \frac{1}{Z}\left(\;\overset{i}{\underset{j=\text{max}(1,i-W)}{\sum }}\overset{\text{min}(n,j+W)}{\underset{k=i+1}{\sum }}\overset{\text{min}(j+C+1,k-1)}{\underset{p=i+1}{\sum }}\right) & \\ & & \;\beta_{\text{SE}}(j,k-1)\cdot \alpha_{\text{S}}(p-1,k-1)\cdot t(\text{SE}\to (\text{Interior})\to \text{S}) & \\ & & \;+\overset{i}{\underset{j=\text{max}(1,i-W)}{\sum }}\overset{\text{min}(n,j+W)}{\underset{k=i+1}{\sum }}\overset{i}{\underset{q=\text{max}(j+4,k-C-1)}{\sum }} & \\ & & \;\left(\beta_{\text{SE}}(j,k-1)\cdot \alpha_{\text{S}}(j,q)\cdot t(\text{SE}\to (\text{Interior})\to \text{S})\right) & \\ p(i,E) & = & \frac{1}{Z}\left(\alpha_{\text{O}}(i-1)\cdot \beta_{\text{O}}(i)\cdot t(\text{O}\to \text{O})\right) & \\ p(i,H) & = & \frac{1}{Z}\overset{i-1}{\underset{j=\text{max}(1,i-W)}{\sum }}\overset{k=\text{min}(n,i+W)}{\underset{k=i+1}{\sum }}\beta_{\text{SE}}(j,k-1)\cdot t(\text{SE}\to (\text{Hairpin})) & \\ p(i,I) & = & \frac{1}{Z}\left(\;\overset{i}{\underset{j=\text{max}(1,i-W)}{\sum }}\overset{\text{min}(n,j+W)}{\underset{k=i+1}{\sum }}\overset{\text{min}(j+C+1,k-1)}{\underset{p=i+1}{\sum }}\overset{k}{\underset{q=\text{max}(p+4,k-C-p+j-1)}{\sum }}\right) & \\ & & \;\beta_{\text{SE}}(j,k-1)\cdot \alpha_{\text{S}}(p-1,q)\cdot t(\text{SE}\to (\text{Interior})\to \text{S}) & \\ & & \;+\overset{i}{\underset{j=\text{max}(1,i-W)}{\sum }}\overset{\text{min}(n,j+W)}{\underset{k=i+1}{\sum }}\overset{\text{min}(j+C+1,i-1)}{\underset{p=j+1}{\sum }}\overset{i}{\underset{q=\text{max}(p+4,k-C-p+j-1)}{\sum }} & \\ & & \;\;\;\;\;\;\left(\beta_{\text{SE}}(j,k-1)\cdot \alpha_{\text{S}}(p-1,q)\cdot t(\text{SE}\to (\text{Interior})\to \text{S})\right) & \\ p(i,M) & = & \frac{1}{Z}\left\{\begin{array}{l} \overset{\text{min}(i+W,n)}{\underset{k=i}{\sum }}\beta_{\text{M}}(i-1,k)\cdot \alpha_{\text{M}}(i,k)\cdot t(\text{M}\to \text{M})\\ \overset{i}{\underset{k=\text{max}(0,i-W)}{\sum }}\beta_{\text{M2}}(i,k)\cdot \alpha_{\text{M2}}(k,i-1)\cdot t(\text{M2}\to \text{M2}) \end{array}\right) & \\ p(i,S) & = & \frac{1}{Z}\overset{\text{min}(n,i+W)}{\underset{j=\text{max}(0,i-W)}{\sum }}\left\{\begin{array}{l} \beta_{\text{S}}(i-1,j)\cdot \alpha_{\text{SE}}(i,j-1)\cdot t(\text{S}\to \text{SE})\\ \beta_{\text{S}}(i-1,j)\cdot \alpha_{\text{S}}(i,j-1)\cdot t(\text{S}\to \text{S}) \end{array}\right) & \end{array}$$

Here, O is the outer state, S is the stem state, SE is the stem-end state, M is the multi state and M2 is the multi2 state in the Rfold model.

### Implementation

We implemented the algorithms in C++ as a program named CapR. CapR exhaustively computes the structural profile {*p*(*i*,*δ*)} for a given RNA sequence with *O*(*NW*^2^) time and *O*(*NW*) memory. We used a portion of the source code from the Vienna RNA package [43]. We include the source code as Additional file 2. Our source code is also available from [44].

### Data preparation and analysis

To evaluate the accuracy of the structural profiles calculated by CapR, we used 188 structural RNA families in the Rfam 10.0 seed dataset [22]. They are provided as 188 structural alignments with experimentally validated pseudoknot-free structures. By excluding alignment columns with a gap proportion of ≥0.5, we obtained 8,775 sequences and 1,039,537 nucleotides.

In the present study, we focused on RBP target recognition. In this application, it should be ineffective to consider transcribed sequences that are too long because regions that are too distant are unlikely to affect the secondary structures around the RBP-bound sites, although our algorithm itself can be applied to long RNAs. Therefore, we investigated how much distance we should take into account. We prepared 100 random RNA sequences 10,100 nucleotides long and truncated them so that the lengths of the flanking sequences of the central 100 bases became *l*=250,500,…, 2,500. Then, we calculated the structural profiles of the central 100 bases for each *l*, and calculated the Pearson correlation coefficient between the structural profiles of the original sequence and those of the truncated sequences. Additional file 1: Figure S8 shows that the Pearson correlation coefficients were more than 0.99 for *l*≥2,000. Therefore, we considered 2,000 nucleotides upstream and downstream of the RBP-bound sites in this study.

To investigate the structural characteristics of RNAs around the RBP-binding sites, we downloaded CLIP-seq datasets from the doRina database [23] (human [45], mouse [46] and nematode [47]). We excluded from the analysis CLIP-seq datasets that met one of the following three criteria: (1) well-defined sequential motifs not presented in the original paper of the dataset, (2) datasets for mutant RBPs and (3) the average number of RBP-bound sites (that is the sequential motif-matched sites within the CLIP-seq peak regions defined in doRina) is less than two. The third criterion was adopted because many RBP-bound sites include false positives. As a result, we selected ten RBPs: GLD-1 (nematode), QKI (human), Pum2 (human), SRSF1 (human), Nova (mouse), Lin28A (mouse), FXR1 (human), FXR2 (human), FMR1_7 (human) and FMR1_1 (human) [7,24-28]. When the peak regions spanned just one or two bases, we sought sequential motif-matched sites within ±10 nucleotides around the peak regions. If no motif-matched sites were found, such peak regions were excluded from the analysis. Then, we extracted ±2,000 nucleotide sequences around the RBP-bound sites to create the positive datasets. If there existed multiple RBP-bound sites in the same peak region, we averaged the structural profiles around those sites and used them as a single observation. For each gene in RefSeq [48], the transcribed sequence was defined by the genomic region between the most upstream 5^′^ position and the most downstream 3^′^ position of its mRNA isoforms. To generate the shuffled and partially shuffled datasets, we used the uShuffle software to preserve the di-nucleotide frequencies of the original sequences [49]. The data sizes and other basic statistics of the CLIP-seq datasets are summarized in Additional file 1: Tables S1 and S2. In the present study, because the distributions of the structural profiles did not follow a normal distribution, we used the non-parametric Wilcoxon–Mann–Whitney test.

We also examined how the choice of the maximal span *W* influences the results. We compared the highest *P* scores of the exterior and multibranch loops with different *W* because these two loops are sensitive to *W*. We calculated the ratios of the *W* sensitivity (*δ*) of the highest *P* scores among all positions for each loop *δ* calculated at *W*=400 and 30:

$$\begin{array}{l} W\text{sensitivity}(\delta )=\frac{\text{Highest}\;\;\text{P}\;\;\text{score for}\;\delta \;\text{at}\;W=400}{\text{Highest}\;\;\text{P}\;\;\text{score for}\;\delta \;\text{at}\;W=30} \end{array}$$

Additional file 1: Figure S9 is a box plot of the *W* sensitivity of the exterior loop, multibranch loop and unstructured contexts for all the RBP datasets. The highest *P* scores of the exterior and multibranch loops were sensitive to *W*, whereas the highest *P* score of unstructured context was insensitive to *W*.

## Notes added in proof

After the manuscript was accepted, we were informed that the similar algorithm to CapR was internally used in the previous researches [50-52].

## Abbreviations

AUROC: Area under the receiver operating characteristic curve; CLIP: Cross-linking immunoprecipitation; iCLIP: Individual-nucleotide resolution CLIP; miRNA: microRNA; PAR-CLIP: Photoactivatable-ribonucleoside-enhanced CLIP; RBP: RNA-binding protein; RIP-Chip: RNA-binding protein immunoprecipitation microarray; SCFG; Stochastic context-free grammar; seq: Sequencing; siRNA: Small interfering RNA; snoRNA: Small nucleolar RNA.

## Competing interests

The authors declare that they have no competing interests.

## Authors’ contributions

TF, KA and HK designed the project. TF and HK developed the algorithm. TF implemented the software and performed the analyses. HO, GT, KA and WI advised on the project. TF, HO, WI and HK wrote the paper. All the authors read and approved the final manuscript.

## Supplementary Material

Additional file 1 **Supplementary materials.** This file includes additional figures and tables not shown in the manuscript. Click here for file

Additional file 2 **The source code of CapR.** This file includes the source code of CapR. Click here for file
