# Supplementary material for: CapR: revealing structural specificities of RNA-binding protein target recognition using CLIP-seq data
Source: Genome Biol. 2014 Jan 21;15(1):R16. doi: 10.1186/gb-2014-15-1-r16 (PMC4053987; doi:10.1186/gb-2014-15-1-r16)
Supplement: Additional file 1 — Supplementary materials. This file includes additional figures and tables not shown in the manuscript. [file gb-2014-15-1-r16-S1.pdf]

Supplementary Tables

Table 1 - Basic statistics of the CLIP-seq datasets

Average number denotes the average number of motif-matched sites within the peak regions.

RBP	motif	species(assembly)	experiment	number of motif site	average number
GLD-1	AYUAAY	C.elegance(ce6)	PAR-CLIP	385	1.17
QKI	AYUAAY	H.sapiens(hg18)	PAR-CLIP	3054	1.26
Pum2	UGUANAUA	H.sapiens(hg18)	PAR-CLIP	1327	1.054
SF2ASF	GAAGAA	H.sapiens(hg18)	HITS-CLIP	2721	1.2521
Nova	YCAY	M.musclus(mm9)	HITS-CLIP	24019	1.345
Lin28A	AAGNNG	M.musclus(mm9))	HITS-CLIP	28642	1.1164
FXR1	ACUK or WGGA	H.sapiens(hg18)	PAR-CLIP	2634	1.15
FXR2	ACUK or WGGA	H.sapiens(hg18)	PAR-CLIP	12886	1.2112
FMR1_7	ACUK or WGGA	H.sapiens(hg18)	PAR-CLIP	46826	1.43478
FMR1_1	ACUK or WGGA	H.sapiens(hg18)	PAR-CLIP	93678	1.616

Table 2 - The numbers of two known sequential motifs for the CLIP-seq data set of the FMRP family

RBP	ACUK	WGGA	Total
FXR1	2435	199	2634
FXR2	9829	3057	12886
FMR1_7	19159	27667	46826
FMR1_1	46364	47314	93678

Supplementary Figures

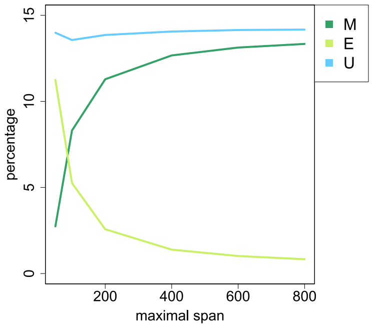

Supplementary Fig.1 Dependence of the exterior loop, multibranch loop, and unstructured context on the maximal span W. The x-axis represents the maximal span W. The y-axis represents the averaged $p(i,\delta)$ over all the nucleotides.

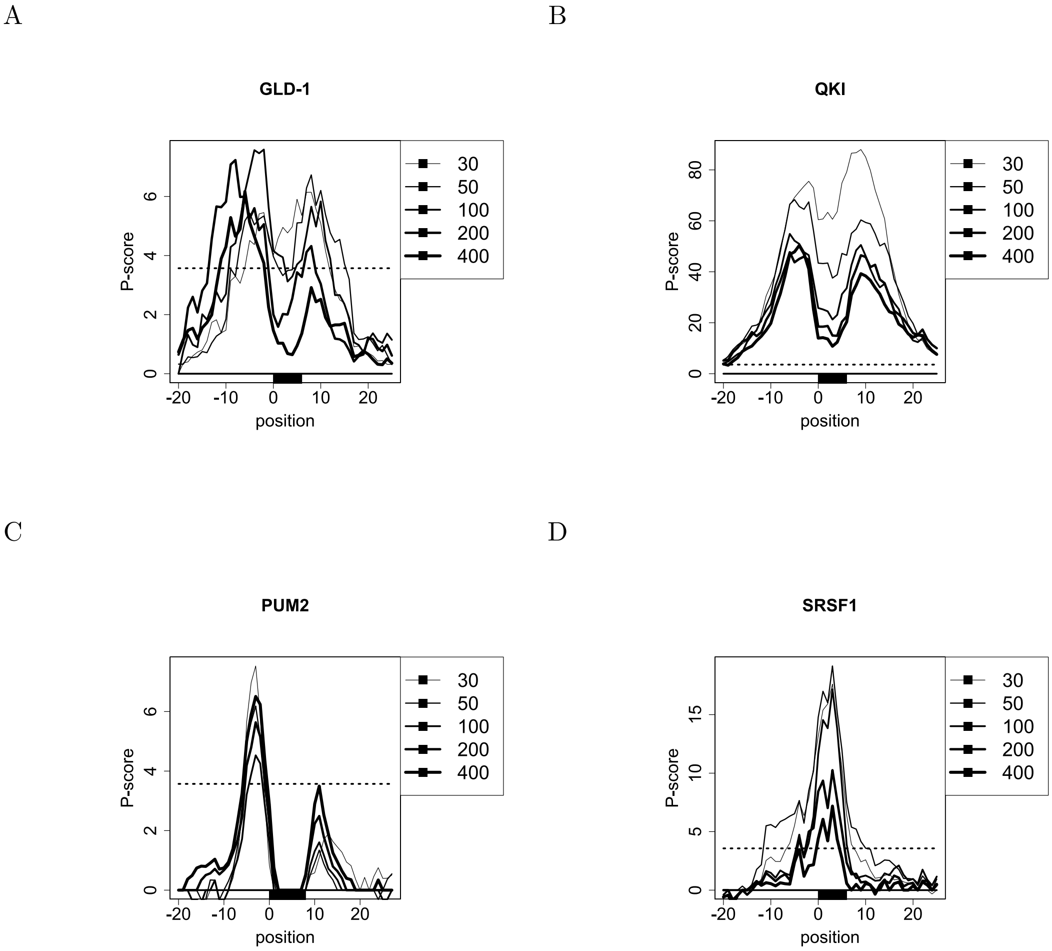

E

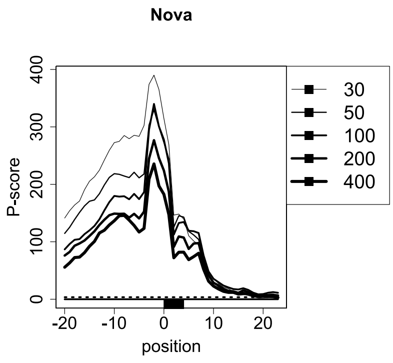
Nova
P-score
position
30
50
100
200
400

F

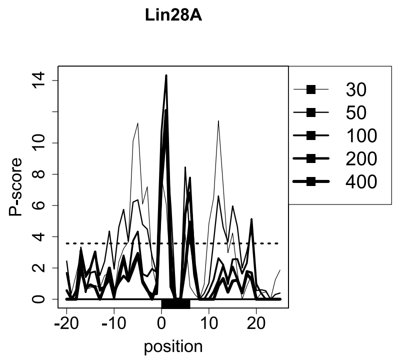
Lin28A
P-score
position
30
50
100
200
400

G

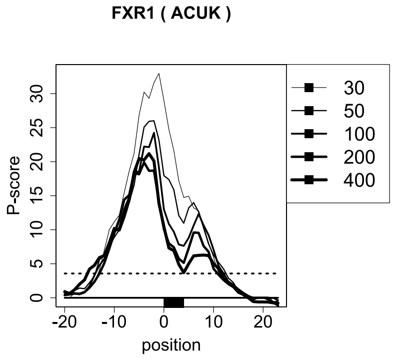
FXR1 (ACUK)
P-score
position
30
50
100
200
400

H

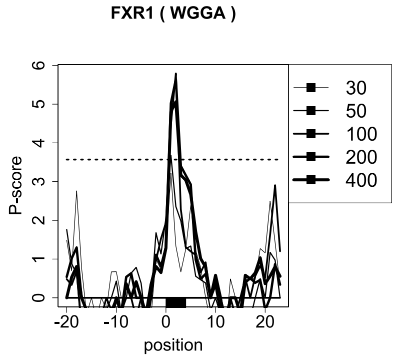
FXR1 (WGGA)
P-score
position
30
50
100
200
400

I

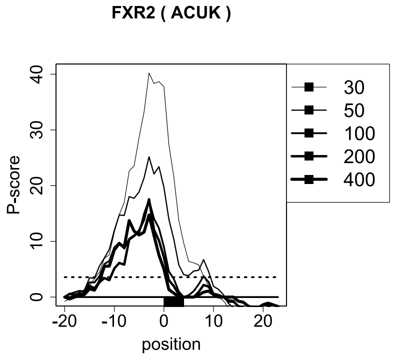
FXR2 (ACUK)
P-score
position
30
50
100
200
400

J

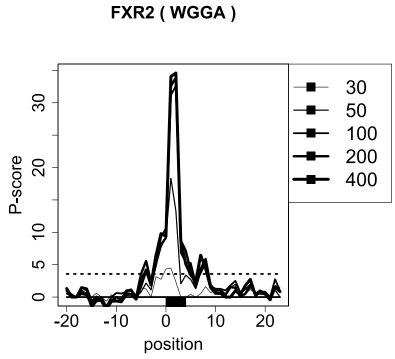
FXR2 (WGGA)
P-score
position
30
50
100
200
400

K

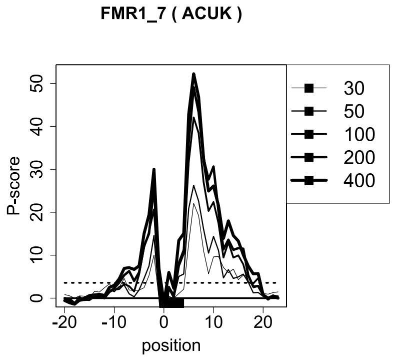
FMR1_7 (ACUK)
P-score
position
30
50
100
200
400

L

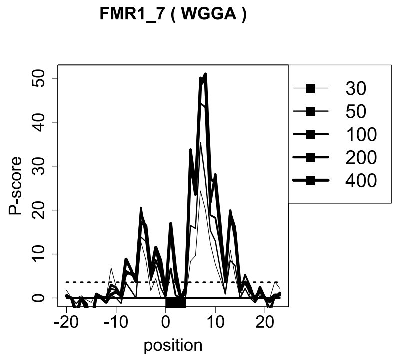
FMR1_7 (WGGA)
P-score
position
30
50
100
200
400

M

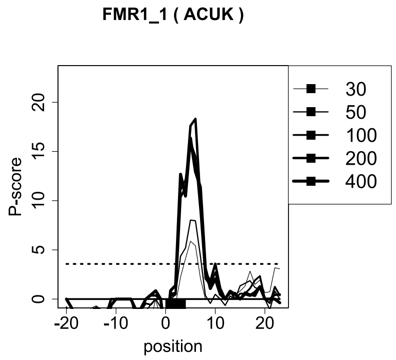

N

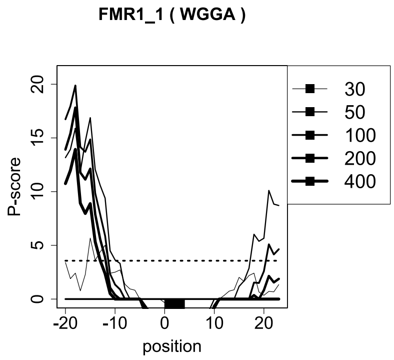

Supplementary Fig.2 Dependence of the distribution of P-score on the maximal span W. The distribution of P-scores with various maximal span W for (A) GLD-1(unstructured), (B) QKI (unstructured), (C) Pum2 (hairpin), (D) SRSF1 (unstructured), (E) Nova (unstructured), (F) Lin28A (internal), (G) FXR1(ACUK) (unstructured), (H) FXR1(WGGA) (stem), (I) FXR2(ACUK) (unstructured), (J) FXR2(WGGA) (stem), (K) FMR1_7(ACUK) (stem), (L) FMR1_7(WGGA) (stem), (M) FMR1_1(ACUK) (stem), and (N) FMR1_1(WGGA) (hairpin). The x-axis represents nucleotide positions. The y-axis is P-score. The black box represents sequential motif sites. For each RBP, only the distribution for structural context with highest P-score at $W = 200$ are shown.

A

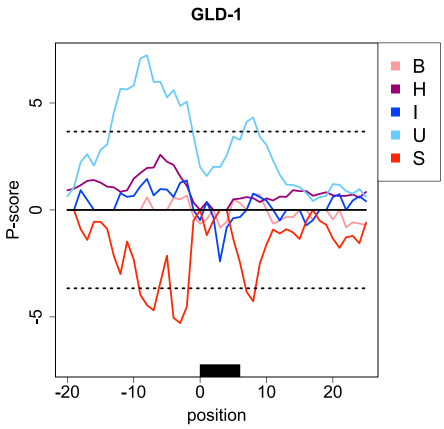

B

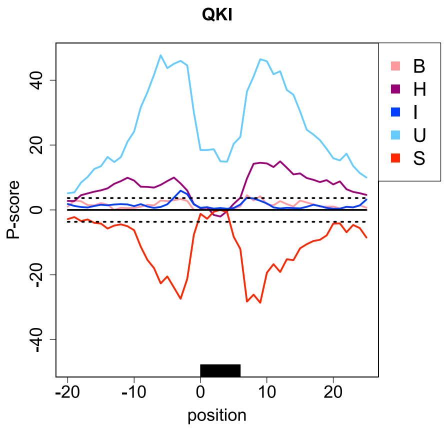

C

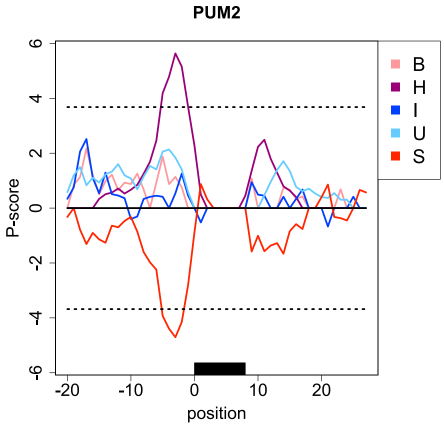

D

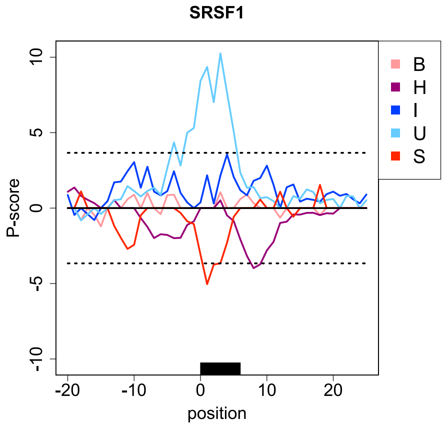

E

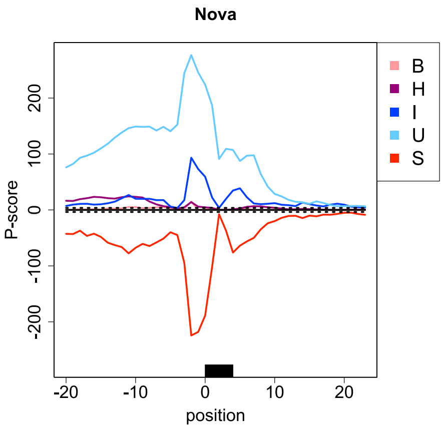
Nova
P-score
position
B
H
I
U
S

F

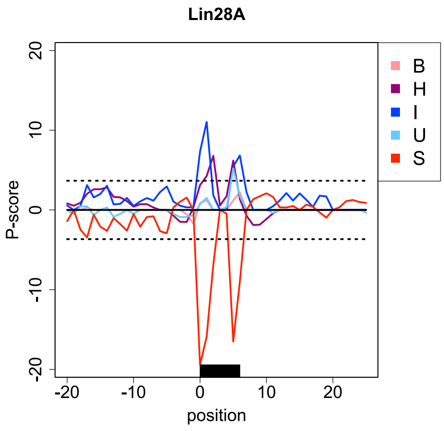
Lin28A
P-score
position
B
H
I
U
S

G

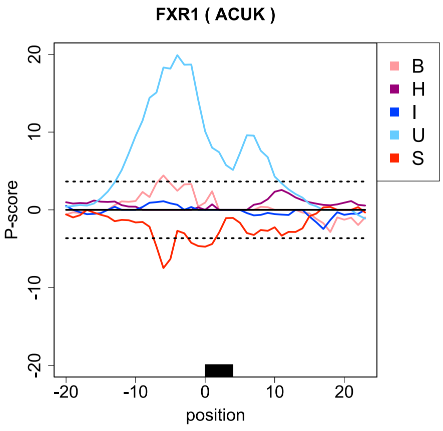
FXR1 (ACUK)
P-score
position
B
H
I
U
S

H

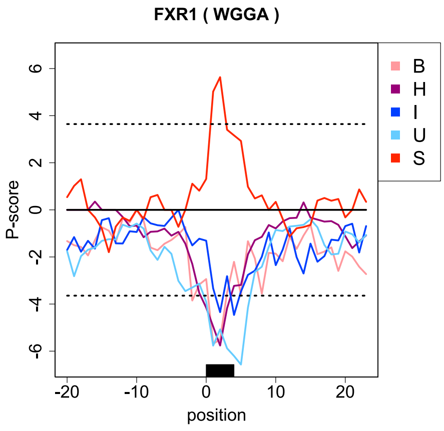
FXR1 (WGGA)
P-score
position
B
H
I
U
S

I

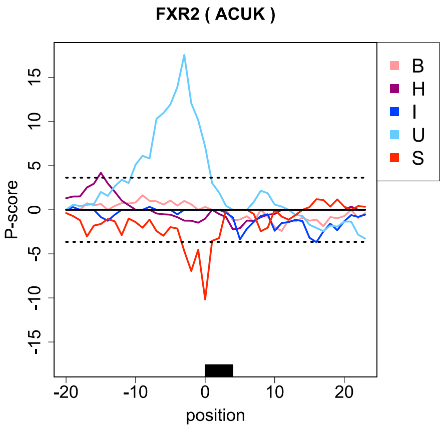
FXR2 (ACUK)
P-score
position
B
H
I
U
S

J

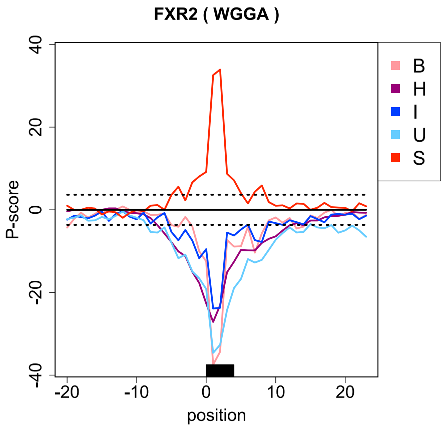
FXR2 (WGGA)
P-score
position
B
H
I
U
S

K

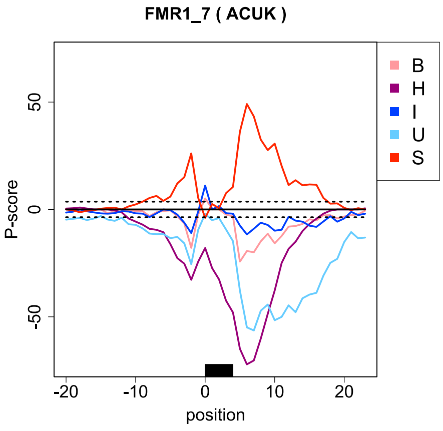
FMR1_7 (ACUK)
P-score
position
B
H
I
U
S

L

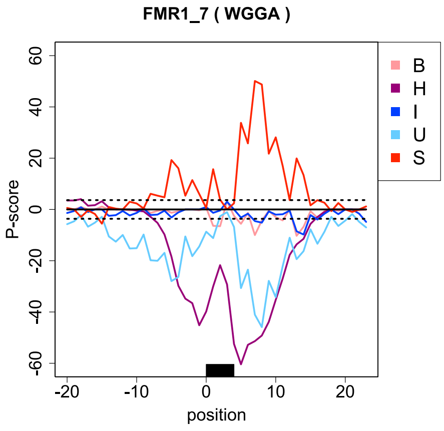
FMR1_7 (WGGA)
P-score
position
B
H
I
U
S

M

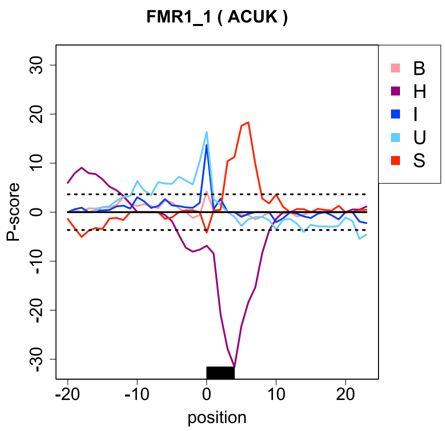

N

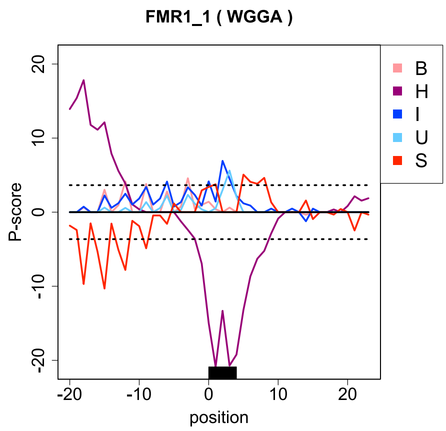

Supplementary Fig.3 The distribution of the P-scores for each RBP. The x-axis represents nucleotide positions, and the y-axis represents P-score of ±20 bases around the sequential motif site. The position 0 denotes the start position of the sequential motif. Positive P-scores for each structural context indicate that the positions tend to prefer the structural context. The black box represents the sequential motif site. The dotted lines are the corrected significance level of Bonferroni correction ($\alpha = 0.05$). Each panel represents the distribution of P-score for (A) GLD-1, (B) QKI, (C) Pum2, (D) SRSF1, (E) Nova, (F) Lin28A, (G) FXR1(ACUK), (H) FXR1(WGGA), (I) FXR2(ACUK), (J) FXR2(WGGA), (K) FMR1_7(ACUK), (L) FMR1_7(WGGA), (M) FMR1_1(ACUK), and (N) FMR1_1(WGGA).

A

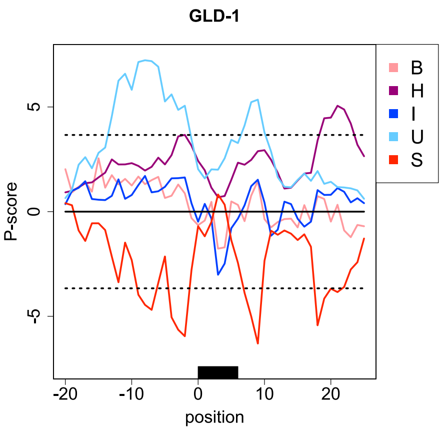

B

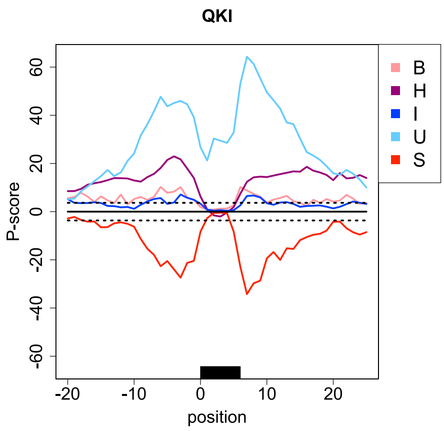

C

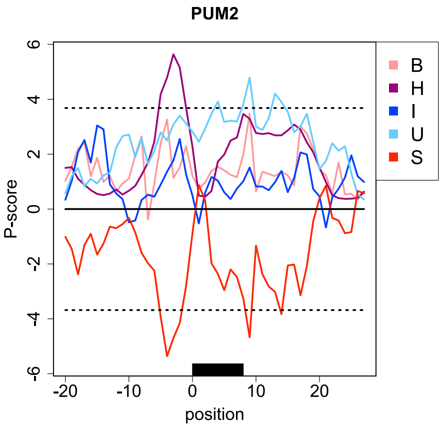

D

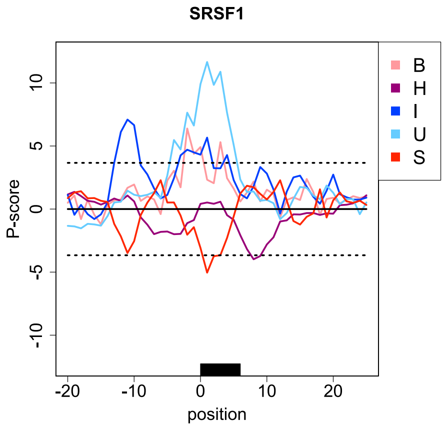

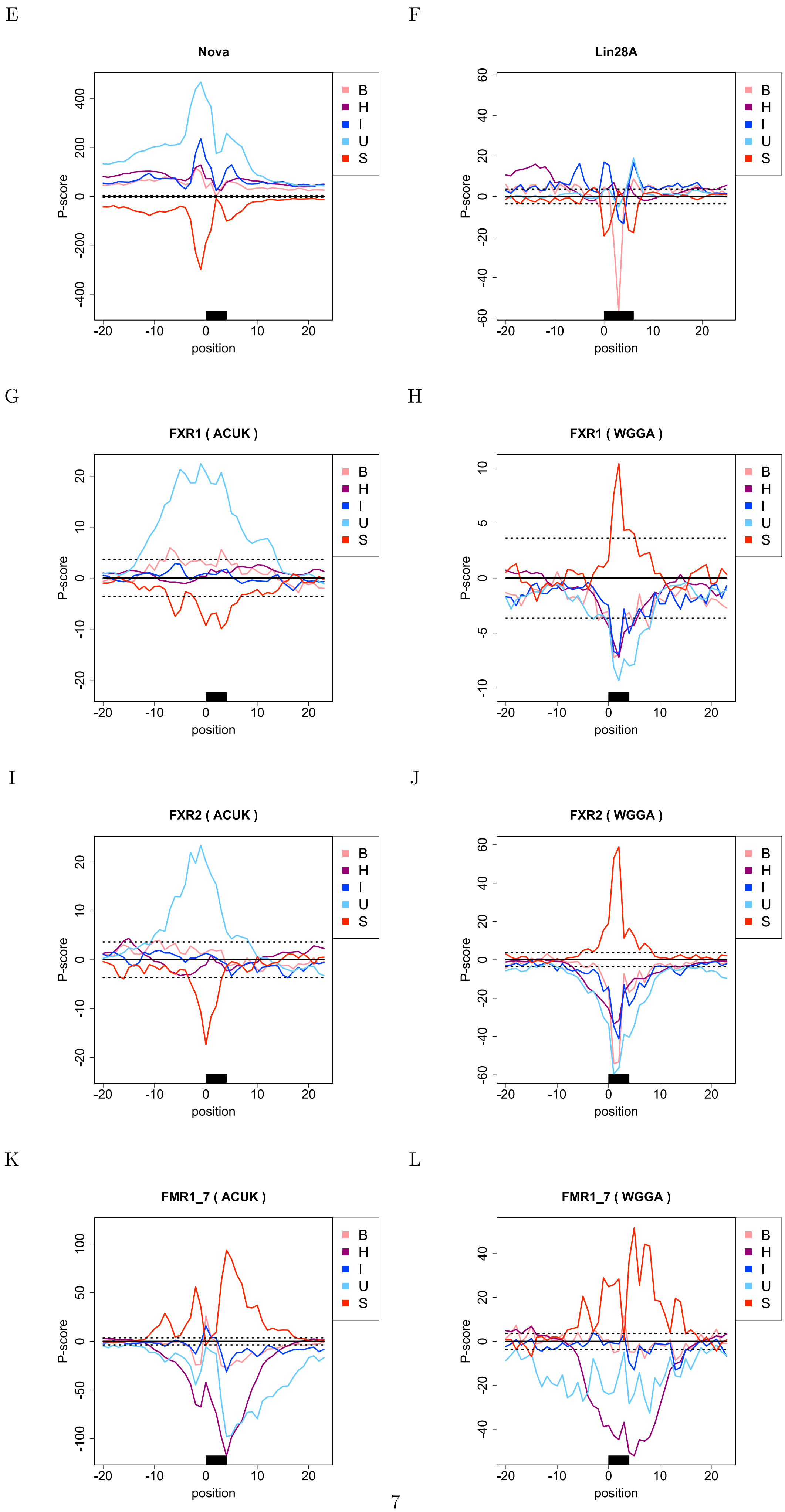
E
Nova
F
Lin28A
G
FXR1 (ACUK)
H
FXR1 (WGGA)
I
FXR2 (ACUK)
J
FXR2 (WGGA)
K
FMR1_7 (ACUK)
L
FMR1_7 (WGGA)
P-score
position
B
H
I
U
S

M

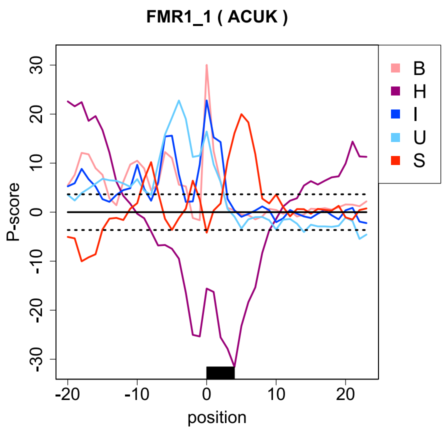

N

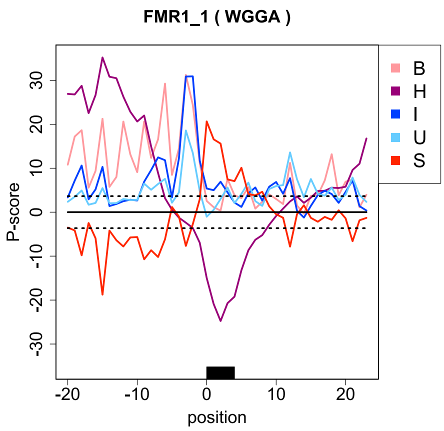

Supplementary Fig.4 The distribution of the P-scores for the unbound datasets. The x-axis represents nucleotide positions, and the y-axis represents P-score of ±20 bases around the sequential motif site. The position 0 denotes the start position of the sequential motif. Positive P-scores for each structural context indicate that the positions tend to prefer the structural context. The black box represents the sequential motif site. The dotted lines are the corrected significance level of Bonferroni correction ($\alpha = 0.05$). Each panel represents the distribution of P-score for (A) GLD-1, (B) QKI, (C) Pum2, (D) SRSF1, (E) Nova, (F) Lin28A, (G) FXR1(ACUK), (H) FXR1(WGGA), (I) FXR2(ACUK), (J) FXR2(WGGA), (K) FMR1_7(ACUK), (L) FMR1_7(WGGA), (M) FMR1_1(ACUK), and (N) FMR1_1(WGGA).

A

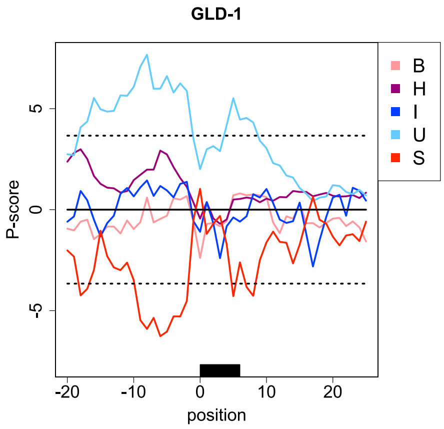

B

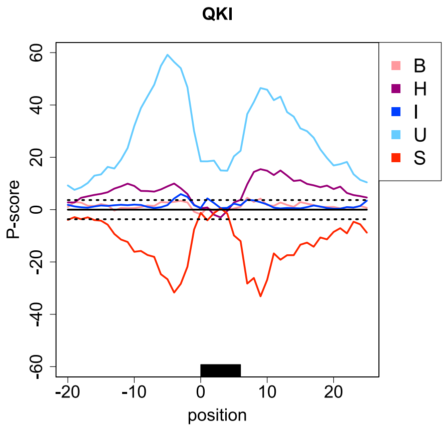

C

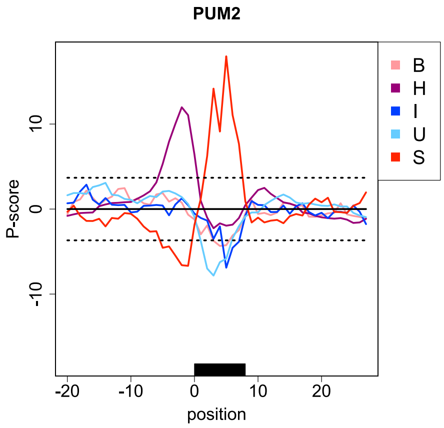

D

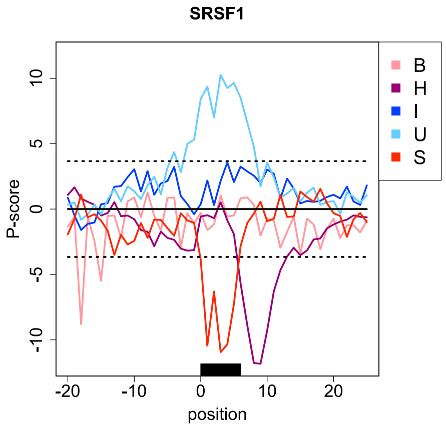

E

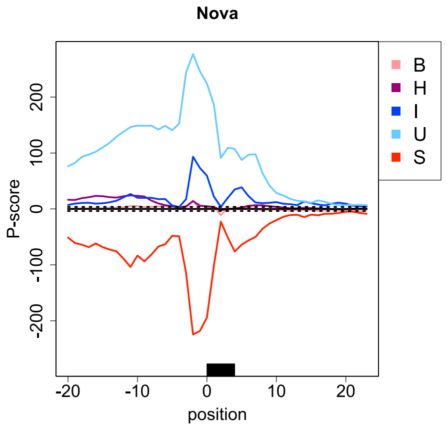
Nova
P-score
position
-20 -10 0 10 20
-200 -100 0 100 200
B
H
I
U
S

F

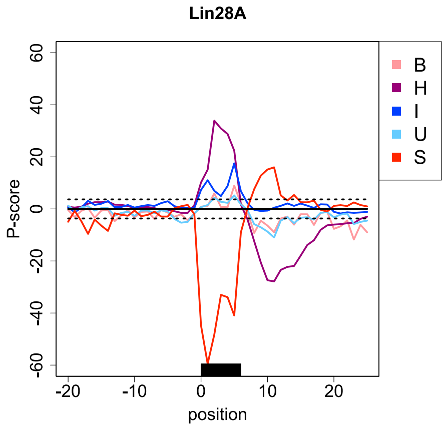
Lin28A
P-score
position
-20 -10 0 10 20
-60 -40 -20 0 20 40 60
B
H
I
U
S

G

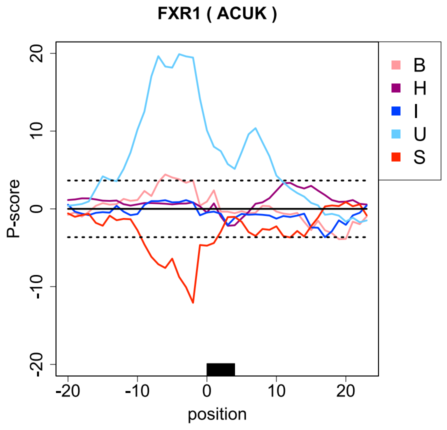
FXR1 (ACUK)
P-score
position
-20 -10 0 10 20
-20 -10 0 10 20
B
H
I
U
S

H

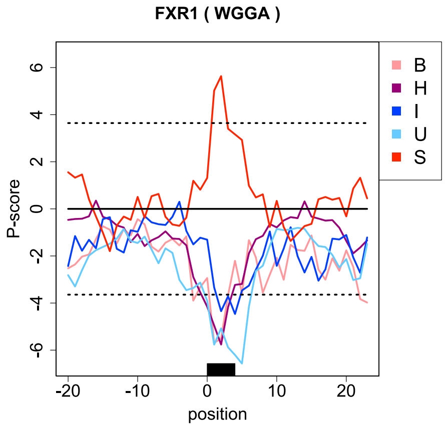
FXR1 (WGGA)
P-score
position
-20 -10 0 10 20
-6 -4 -2 0 2 4 6
B
H
I
U
S

I

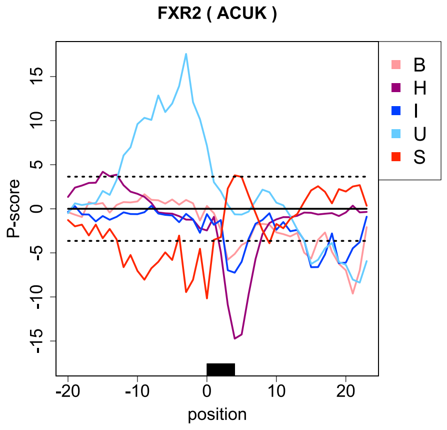
FXR2 (ACUK)
P-score
position
-20 -10 0 10 20
-15 -10 -5 0 5 10 15
B
H
I
U
S

J

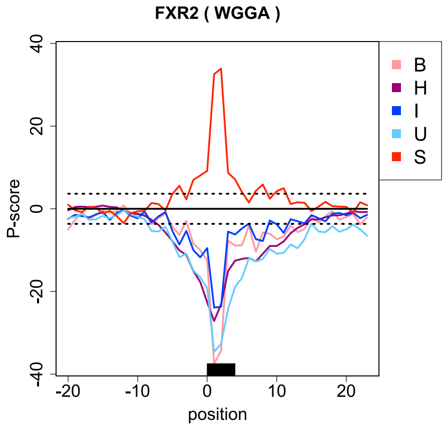
FXR2 (WGGA)
P-score
position
-20 -10 0 10 20
-40 -20 0 20 40
B
H
I
U
S

K

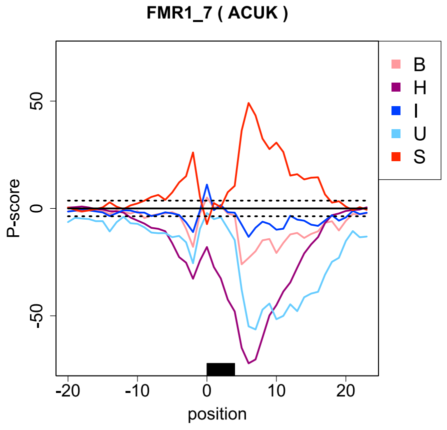
FMR1_7 (ACUK)
P-score
position
-20 -10 0 10 20
-50 0 50
B
H
I
U
S

L

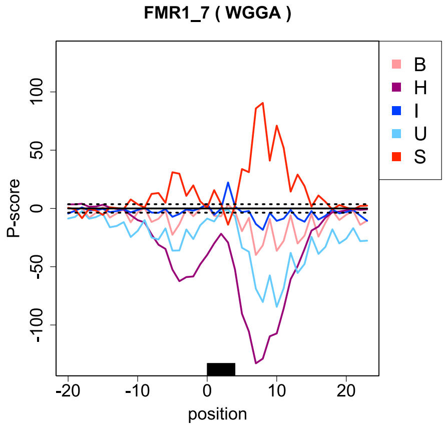
FMR1_7 (WGGA)
P-score
position
-20 -10 0 10 20
-100 -50 0 50 100
B
H
I
U
S

M

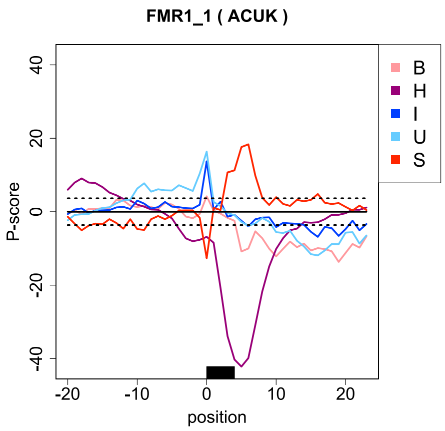

N

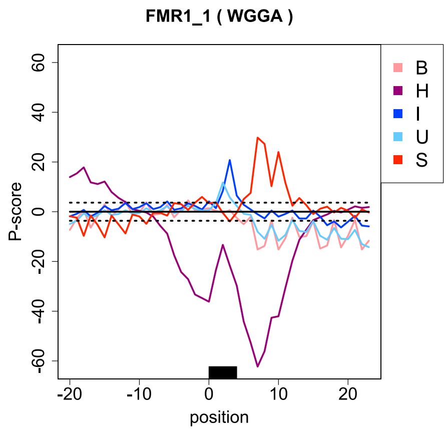

Supplementary Fig.5 The distribution of the P-scores for the shuffled datasets. The x-axis represents nucleotide positions, and the y-axis represents P-score of ±20 bases around the sequential motif site. The position 0 denotes the start position of the sequential motif. Positive P-scores for each structural context indicate that the positions tend to prefer the structural context. The black box represents the sequential motif site. The dotted lines are the corrected significance level of Bonferroni correction ($\alpha = 0.05$). Each panel represents the distribution of P-score for (A) GLD-1, (B) QKI, (C) Pum2, (D) SRSF1, (E) Nova, (F) Lin28A, (G) FXR1(ACUK), (H) FXR1(WGGA), (I) FXR2(ACUK), (J) FXR2(WGGA), (K) FMR1_7(ACUK), (L) FMR1_7(WGGA), (M) FMR1_1(ACUK), and (N) FMR1_1(WGGA).

A

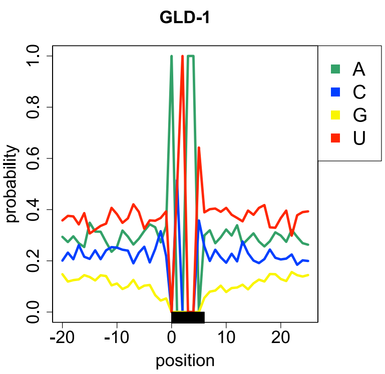

B

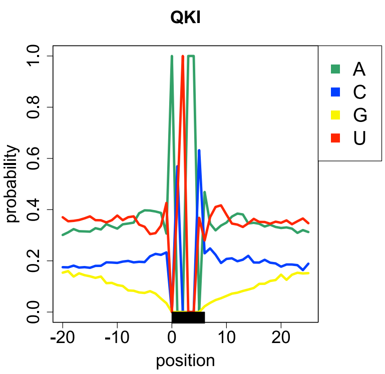

C

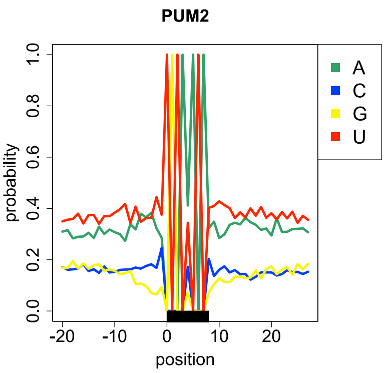

D

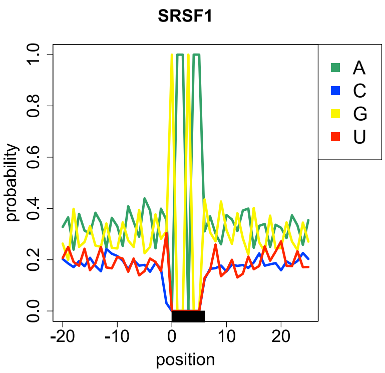

E

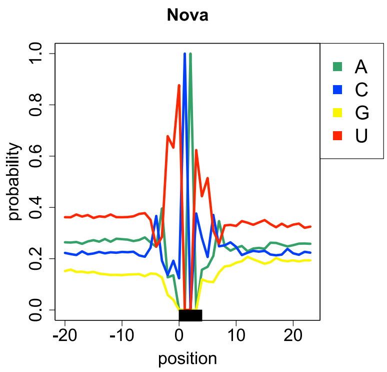
Nova
probability
position
A
C
G
U

F

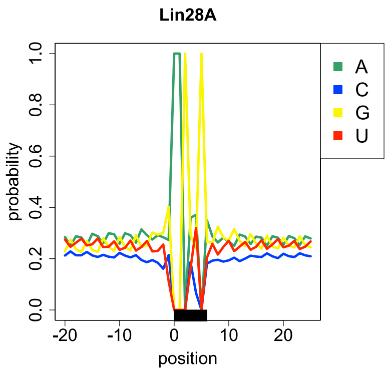
Lin28A
probability
position
A
C
G
U

G

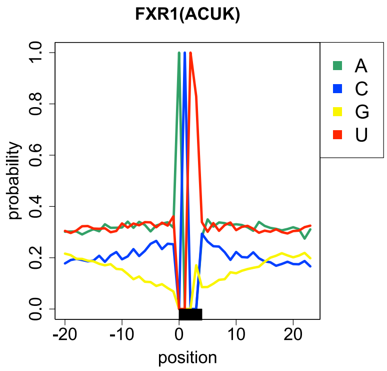
FXR1(ACUK)
probability
position
A
C
G
U

H

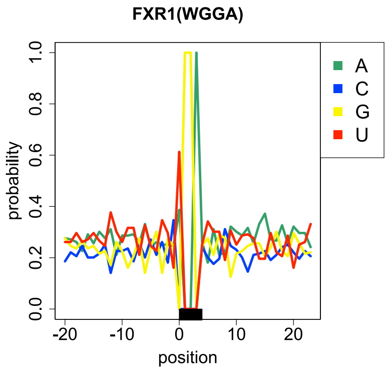
FXR1(WGGA)
probability
position
A
C
G
U

I

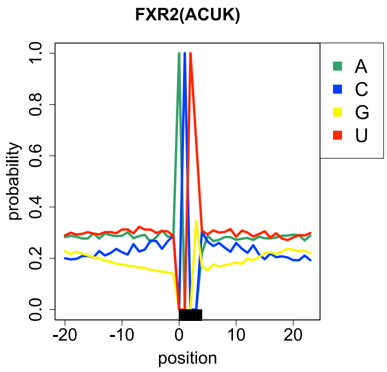
FXR2(ACUK)
probability
position
A
C
G
U

J

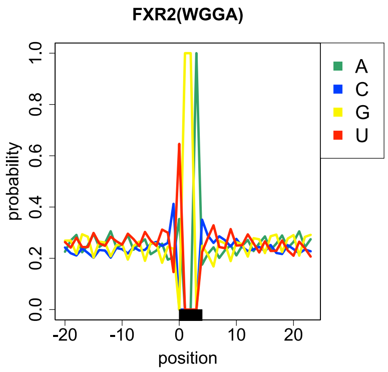
FXR2(WGGA)
probability
position
A
C
G
U

K

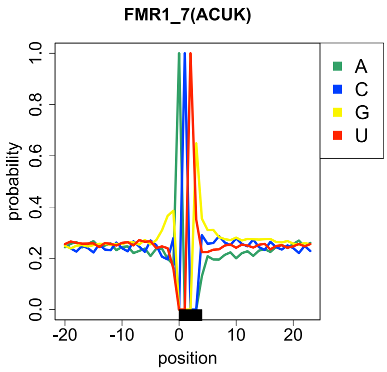
FMR1_7(ACUK)
probability
position
A
C
G
U

L

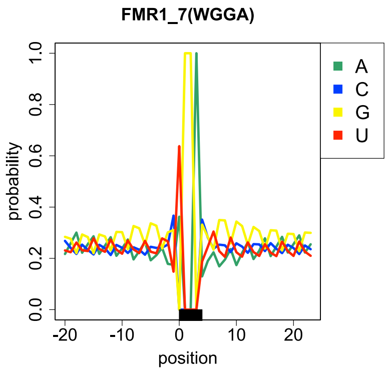
FMR1_7(WGGA)
probability
position
A
C
G
U

M

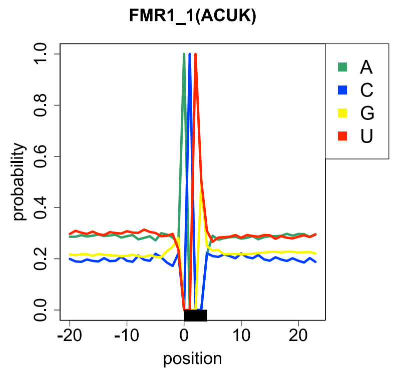

N

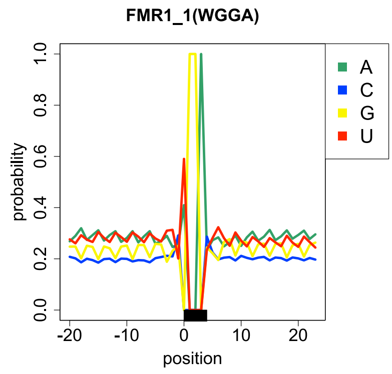

Supplementary Fig.6 The nucleotide composition around the each RBP-bound sites. The nucleotide compositions of ±20 bases around the RBP-bound sites for (A) GLD-1, (B) QKI, (C) Pum2, (D) SRSF1, (E) Nova, (F) Lin28A, (G) FXR1(ACUK), (H) FXR1(WGGA), (I) FXR2(ACUK), (J) FXR2(WGGA), (K) FMR1_7(ACUK), (L) FMR1_7(WGGA), (M) FMR1_1(ACUK), and (N) FMR1_1(WGGA). The x-axis represents the nucleotide position, and the y-axis is the proportion of each nucleotide. The black box represents the sequential motif site.

A

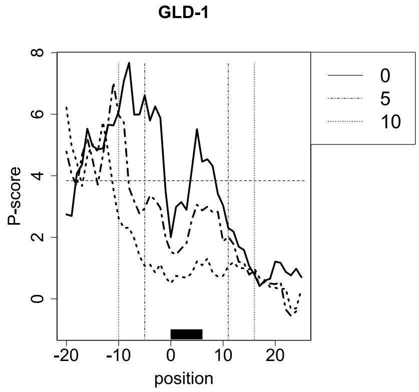

B

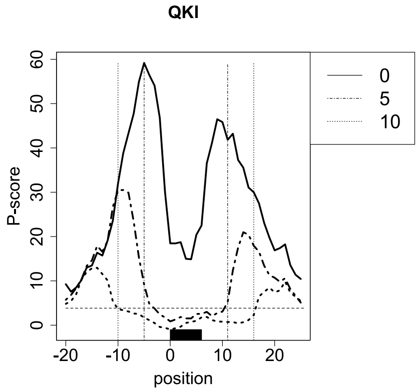

C

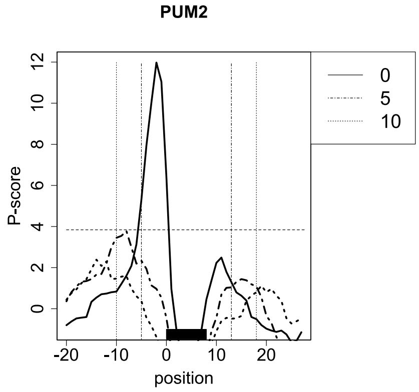

D

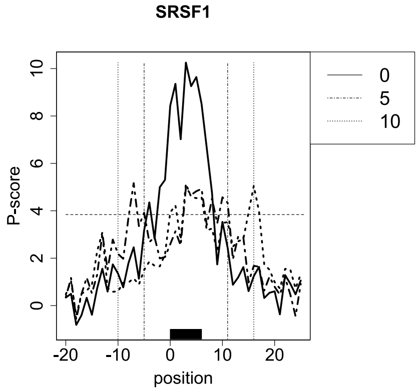

E

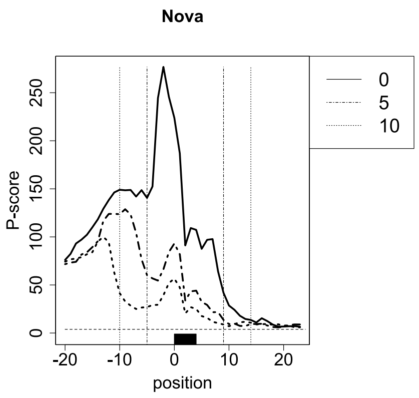
Nova
P-score
position
0
5
10

F

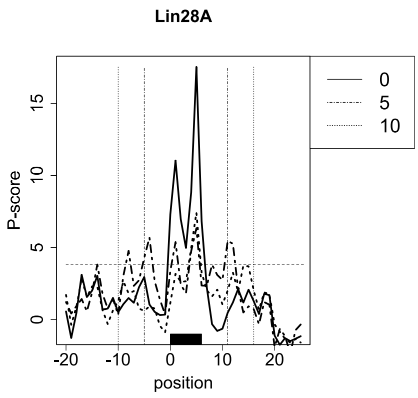
Lin28A
P-score
position
0
5
10

G

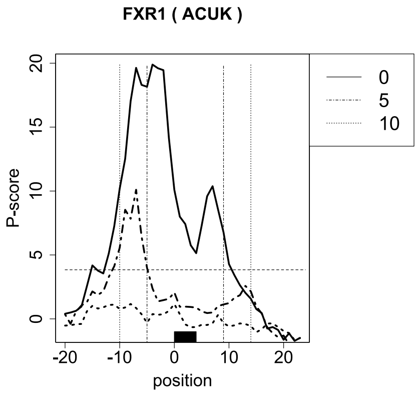
FXR1 (ACUK)
P-score
position
0
5
10

H

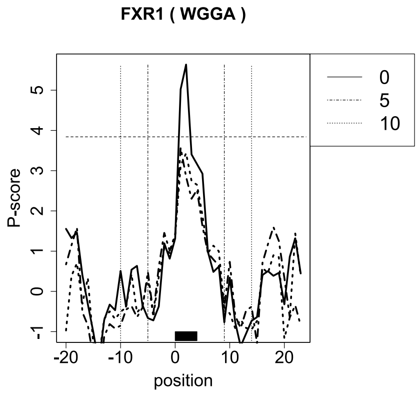
FXR1 (WGGA)
P-score
position
0
5
10

I

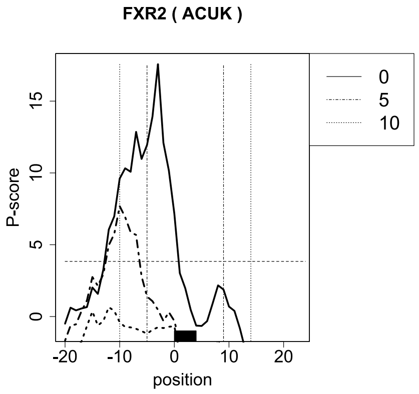
FXR2 (ACUK)
P-score
position
0
5
10

J

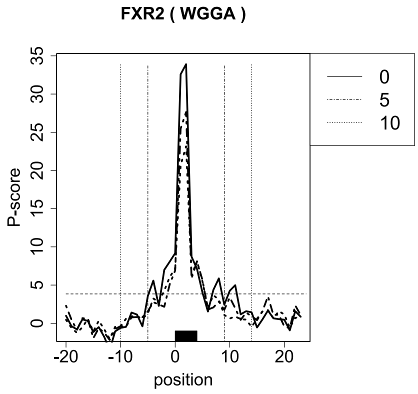
FXR2 (WGGA)
P-score
position
0
5
10

K

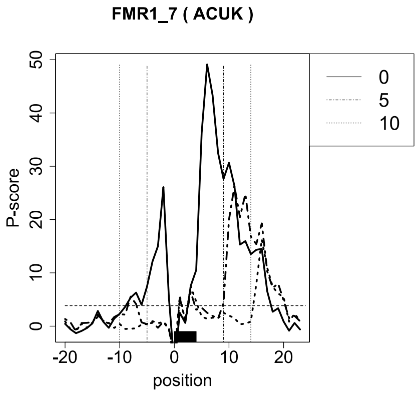
FMR1_7 (ACUK)
P-score
position
0
5
10

L

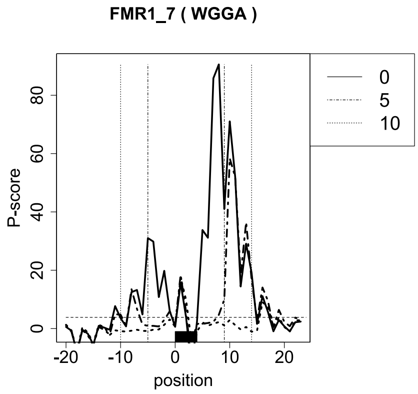
FMR1_7 (WGGA)
P-score
position
0
5
10

M N

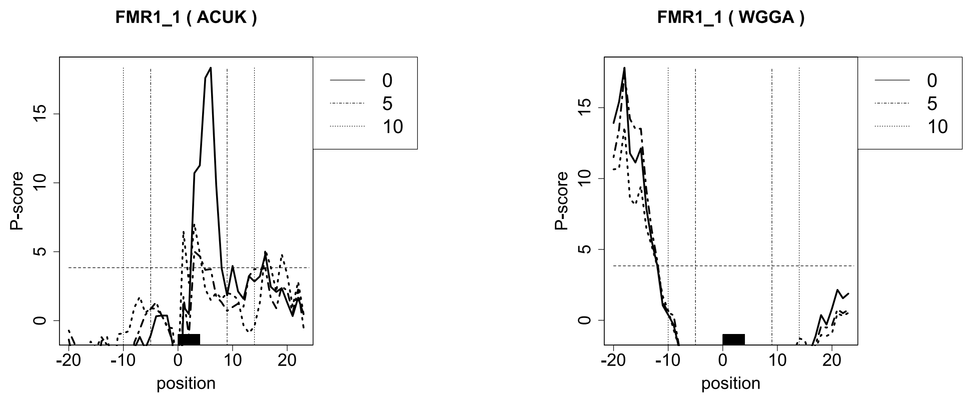

Supplementary Fig.7 The comparison of P-scores of the positive datasets with P-scores of the shuffled and partially shuffled datasets. The shuffled, the partially shuffled (±5), and the partially shuffled (±10) datasets are represented by 0, 5, and 10, respectively. The x-axis represents the nucleotide position, and the y-axis represents P-score of (A) GLD-1(unstructured), (B) QKI (unstructured), (C) Pum2 (hairpin), (D) SRSF1 (unstructured), (E) Nova (unstructured), (F) Lin28A (internal), (G) FXR1(ACUK) (unstructured), (H) FXR1(WGGA) (stem), (I) FXR2(ACUK) (unstructured), (J) FXR2(WGGA) (stem), (K) FMR1_7(ACUK) (stem), (L) FMR1_7(WGGA) (stem), (M) FMR1_1(ACUK) (stem), and (N) FMR1_1(WGGA) (hairpin). The black box is the RBP-bound sites, and the horizontal dotted line the corrected significance level of Bonferroni correction. The vertical dotted lines indicate the ±5 or 10 nt of RBP-bound sites. Note that, only the distribution for structural context with highest P-score are shown for each RBP.

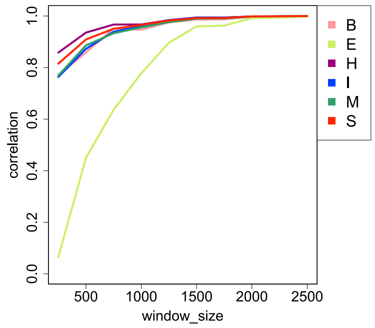

Supplementary Fig.8 The dependence of structural profiles on the truncated length. The x-axis represents the truncated length. The y-axis represents the Pearson correlation coefficient between the structural profiles of the original sequence and those of the truncated sequences.

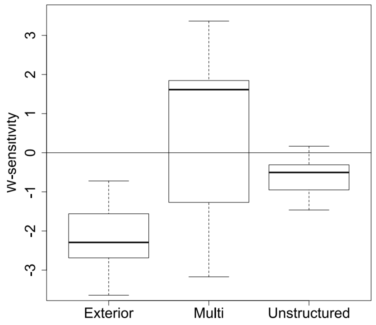

Supplementary Fig.9 The W-sensitivities of exterior loop, multibranch loop, and unstructured contexts for CLIP-seq datasets. The y-axis represents the W-sensitivity. The low W-sensitivity means that the highest P-score at $W = 30$ is larger than that at $W = 400$, and vice versa. When W-sensitivity (δ) equals zero, the structural context δ is completely insensitive to the maximal span.
